# A generative language model decodes contextual constraints on codon choice for mRNA design

**DOI:** 10.1101/2025.05.13.653614

**Published:** 2025-05-13

**Authors:** Marjan Faizi, Helen Sakharova, Liana F. Lareau

**Affiliations:** 1 California Institute for Quantitative Biosciences, University of California, Berkeley, CA 94720, USA; 2 Center for Computational Biology, University of California, Berkeley, CA 94720, USA; 3 Department of Bioengineering, University of California, Berkeley, CA 94720, USA; 4 Chan Zuckerberg Biohub, San Francisco, CA 94158, USA

## Abstract

The genetic code allows multiple synonymous codons to encode the same amino acid, creating a vast sequence space for protein-coding regions. Codon choice can have dramatic implications on mRNA function and protein output, and the impact of these choices has become newly relevant with advances in mRNA technology for vaccines and therapeutics. Genomes have evolved to prefer some codons over others, but simple methods for codon optimization that select the most preferred codons fail to capture complex contextual patterns in natural sequences. To enable biologically informed design of synthetic coding sequences, we present Trias, an encoder-decoder language model trained on the coding regions of millions of eukaryotic mRNA sequences. Trias learns codon usage rules directly from sequence data, integrating local and global dependencies to generate species-specific codon sequences that align with biological constraints. With no explicit training on protein expression, Trias can generate sequences and compute scores that correlate strongly with experimental measurements of mRNA stability, ribosome load, and protein output. The model outperforms commercial codon optimization tools in generating sequences resembling high-expression codon sequence variants. By capturing natural constraints on codon usage within sequence context, Trias provides a powerful, data-driven framework for synthetic mRNA design and for understanding the molecular and evolutionary principles governing codon choice.

## 1 Introduction

Synthetic messenger RNA technology has emerged as a transformative tool for biotechnology, enabling precise control over protein production in living cells and opening new frontiers in synthetic biology, genome engineering, and medicine. mRNAs encoding proteins of interest can be delivered directly into cells, where they are translated into functional proteins using the cell’s own machinery. However, a key challenge remains: designing optimal mRNA sequences to produce heterologous proteins at high levels. Natural mRNAs have many overlapping constraints on their sequences, all necessary for their proper expression and function: the coding region must encode the proper protein with the right fidelity and at the right expression level; the 5^′^ untranslated region must enable translation initiation at the correct rate; and diverse aspects of the sequence determine post-transcriptional regulation, structural stability, and half-life of an mRNA. Several groups have made significant progress in understanding and optimizing UTR elements for synthetic mRNAs (Sample et al., 2019; Lewis et al., 2024). RNA structure and half-life has been another important avenue for optimization (Leppek et al., 2022; Zhang et al., 2023). Here, we focus specifically on the coding sequence and the challenge of codon optimization.

While the desired protein sequence may be pre-determined, there are many ways to encode the same protein sequence in mRNA. The genetic code is degenerate, with 64 possible 3-nucleotide codons specifying only 20 amino acids and three translation stop signals. Most amino acids can be specified by two or more synonymous codons, so mapping a protein sequence back to its codon sequence is non-unique and codon optimization becomes a combinatorially complex problem. For example, the SARS-CoV-2 spike protein has 1,273 amino acids and can be encoded by more than 10^632^ different codon sequences (Kim et al., 2024). Despite this vast number of possibilities, codon choice is not random. Generally, a given organism prefers certain codons over others, with rules that vary across species, but the underlying rules determining the ideal codon for a particular position are complex and not yet understood (Wu and Bazzini, 2023).

The impact of codon choice begins with the basic process of translation: as a ribosome moves along an mRNA, it must wait for matching tRNAs to arrive and decode each codon. Codons recognized by abundant tRNAs tend to be used more frequently and are decoded faster, while those with less abundant tRNAs are used less often and decoded more slowly (Liu, 2020). Groundbreaking work has established that codon choice profoundly affects protein output through multiple mechanisms. Slow translation can destabilize mRNAs through a process termed codon optimality mediated decay (Presnyak et al., 2015; Bazzini et al., 2016; Narula et al., 2019). Slower elongation also directly reduces the number of proteins made per mRNA, even after accounting for differences in stability (Tunney et al., 2018; Barrington et al., 2023; Lyons et al., 2024). We found that codon choice alone could create an eight-fold range in protein output (Tunney et al., 2018). However, maximizing translation speed is not always optimal. Larger multi-domain proteins can require precisely timed translation for proper folding and function (Kim et al., 2015; Yu et al., 2015). Studies of disease-relevant proteins highlight this complexity: faster translation can actually lead to misfolding and reduced protein function (Kimchi-Sarfaty et al., 2007; Kirchner et al., 2017). These differences in translation dynamics influence protein output, co-translational folding, and mRNA stability (Walsh et al., 2020; Narula et al., 2019), collectively influencing the expression of functional proteins. Hence, generating codon sequences that enhance protein expression is challenging but has important applications for biotechnology and therapeutics (Lin et al., 2023a).

These observations underscore the need for sophisticated approaches to sequence design that consider biological context. The classic approach to codon optimization predicts codon sequences for a given protein by leveraging host-specific codon preferences. These methods, implemented in widely used commercial codon optimization tools such as those from GenScript^1^, Twist Biosciences^2^ or IDT^3^, aim to enhance heterologous protein expression by addressing factors such as codon frequency, mRNA secondary structures, or GC content. Most of these approaches rely on manually defined features and cannot fully capture the dependencies between codons. While GenScript employs a more sophisticated machine learning approach, it operates on fragmented sequences, limiting its ability to model long-range dependencies. As a result, these methods lack the capacity to capture complex codon usage rules that span both local and global context across the full codon sequence of a gene. In parallel to these feature-based methods, early deep learning approaches were developed for codon optimization by learning directly from experimental data (Tunney et al., 2018; Jain et al., 2023). These models represented a major step forward by learning codon patterns from functional measurements, but they are based on experimentally derived data that may not be available for all scenarios, and they may not capture complex dependencies in sequences.

Recent advances in machine learning, particularly large language models (LLMs), have introduced powerful data-driven approaches that capture complex information from sequences and bypass the need for explicit prior knowledge or experimental measurements. These models are trained in a self-supervised manner on millions of biological sequences to learn the underlying distributions and patterns of naturally occurring sequences (Simon et al., 2024). LLMs can serve as foundation models that generalize across a wide range of biological tasks, either in a zero-shot setting (without fine-tuning or task-specific examples) or after fine-tuning. For example, the ESM protein foundation models have been trained on millions of protein sequences and fine-tuned for protein structure prediction tasks (Rives et al., 2021; Lin et al., 2023b). Similarly, the Evo genomic foundation models have been trained on millions of DNA sequences and fine-tuned for tasks ranging from variant effect prediction to the generation of novel CRISPR-Cas systems (Nguyen et al., 2024; Brixi et al., 2025).

Building on the success of these large language models, several codon language models have recently been developed and trained on large datasets of mRNA coding sequences (Li et al., 2024; Constant et al., 2023; Outeiral and Deane, 2024; Sidi et al., 2024; Fallahpour et al., 2025; Naghipourfar et al., 2024). These data-driven models have the potential to uncover complex codon usage rules that traditional methods may not capture, providing an improved tool for codon optimization and a framework for studying synonymous codon choice. However, their use for synthetic mRNA design raises a fundamental question, as these models may not align perfectly with the optimization tasks to which they are applied. Natural mRNA sequences are optimized by evolution for their functional need, not necessarily for maximal expression. Many proteins must be made at lower abundance for proper cellular function, and this could create selective pressure for less-optimal codons or, at minimum, a lack of selective pressure for optimal codons. Further, the functional constraints on coding sequences reflect overlapping functional needs of many steps of gene expression, including chromatin accessibility, regulatory elements, and alternative splicing (Liu et al., 2021). Therefore, language models trained on naturally occurring sequences may capture nuanced constraints necessary for proper function of synthetic mRNAs, but this does not guarantee the generation of codon sequences with high protein expression.

Most existing codon language models are predictive and based on encoder-only architectures that are fine-tuned to estimate sequence-level properties. For example, CodonBERT (Li et al., 2024) and CO-BERTa (Constant et al., 2023) are trained to predict features such as mRNA half-life or protein expression to support the design of codon sequences for recombinant protein production or mRNA-based therapeutics. Whereas encoder-only models are well suited for classification and regression tasks, encoder-decoder and decoder-only models are autoregressive architectures designed for sequence generation. Encoder-decoder models first map the input sequence into a latent representation, which is then decoded token by token into the target sequence (Cho et al., 2014). Decoder-only models, on the other hand, use the input as a prompt and generate tokens autoregressively without an intermediate representation. A few codon models have explored generative capabilities. For instance, in addition to their encoder-only model, Constant et al. (2023) also introduced an encoder-decoder model to reverse-translate protein sequences into codons for *E. coli*, although the model showed no improvement over commercial tools in terms of protein expression. Sidi et al. (2024) trained an encoder-decoder model on short fragments (10–75 codons) from just two bacterial and two yeast genomes, limiting the model’s ability to capture long-range dependencies across full-length coding sequences. Naghipourfar et al. (2024) presented EnCodon (encoder-only) and DeCodon (decoder-only), both developed for zero-shot prediction of synonymous variant effects or fine-tuned for tasks such as mRNA half-life and protein expression prediction, but neither model was designed to generate codon sequences for a given protein.

Here, we present Trias, a generative language model based on an encoder-decoder architecture, trained on the full-length coding regions of 10 million eukaryotic mRNA sequences for the task of reverse-translating protein sequences into codon sequences. Unlike all previously published codon language models that were trained on either prokaryotic sequences or a mixture of prokaryotic and eukaryotic sequences, we focus exclusively on eukaryotic mRNA sequences. This focus is due to differences in post-transcriptional regulation between prokaryotes and eukaryotes and enables the model to capture codon usage patterns that are relevant for eukaryotic systems.

We show that Trias captures codon usage patterns that align with biological constraints by learning to generate coding sequences one codon at a time, without being provided a codon table or handcrafted rules to guide the generation process. The model favors frequent codons while preserving rare codons in functionally relevant contexts, particularly in nucleic acid binding genes. Its predictions of rare codons are influenced by sequence context, including adjacent codons and position within the sequence, demonstrating its ability to learn codon usage preferences beyond simple frequency-based heuristics. With no training on experimental data, Trias was able to predict scores for 30 synonymous variants of GFP that correlated strongly with measurements of their protein expression, ribosome load, and mRNA half-life in human cells. Notably, its top-ranked GFP sequence matched high-expression GFP variants more closely than sequences from commercial optimization tools.

Together, these findings highlight the potential of Trias as a data-driven alternative for codon optimization in recombinant protein production and mRNA-based therapeutics, as well as a tool for studying how codon usage is shaped by sequence context. To facilitate its broad application, we have released Trias on Hugging Face, where it enables reverse translation of protein sequences up to 2,046 amino acids in length into codon sequences for over 600 vertebrate species.

## 2 Results

### 2.1 Trias learns codon preferences and amino acid properties through generative training

**Figure 1:**
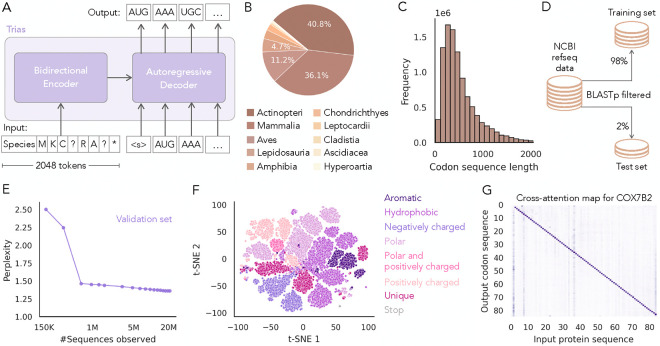
Model overview, dataset composition and training performance. (A) Trias is an encoder-decoder model and reverse translates protein sequences into codon sequences while incorporating species-specific codon preferences. During training, 15% of input tokens are randomly masked (indicated as ‘?’). (B) The model was trained on 10M codon sequences from 640 vertebrate species. (C) Distribution of codon sequence lengths in the dataset. (D) An initial test set was chosen at random and sequences with protein similarity to the initial test set were moved from the training to test set. To monitor training, we additionally randomly sampled 1% of the training data as a validation set. (E) Perplexity during training, showing a decrease from 2.5 to below 1.5 on the validation set. (F) Projection of the final encoder embedding layer for 20 randomly selected human protein sequences, with each dot representing an amino acid. (G) Cross-attention map for example gene, showing how each codon attends to the input amino acid sequence and the species tag.

Trias is a sequence-to-sequence model designed to reverse translate protein sequences into codon sequences (Figure 1A). It is based on the BART architecture, which includes bidirectional encoder and autoregressive decoder modules and is commonly used in natural language processing tasks such as machine translation and text summarization (Lewis et al., 2019). The bidirectional encoder module of Trias processes the entire input protein sequence at once, capturing long-range dependencies between amino acids. It produces a latent representation of the sequence, which is passed to the decoder. The autoregressive decoder then generates the corresponding codon sequence token by token, conditioning each predicted codon on the previously generated codons and the encoder’s representation. To allow the model to learn species-specific preferences for codon usage, we prepend species tags to the protein input sequence. During training, 15% of the tokens from the input protein sequences are randomly masked.

Trias has 47 million parameters and was trained on 10 million sequences from 640 vertebrate species, all sourced from the NCBI RefSeq database (Figure 1B). Each sequence in the dataset can be up to 2,046 tokens long (Figure 1C), with each token representing either an amino acid (input) or a codon (output). This sequence length exceeds the capacity of earlier codon models such as CodonBERT and CaLM. To ensure robust evaluation and prevent information leakage between the training and test sets, we removed homologous sequences from the training data based on protein-level similarity using BLAST(Figure 1D). While Trias is designed to learn codon usage patterns, highly similar sequences in both sets could inflate performance, making it unclear whether the model is truly learning generalizable codon preferences or simply leveraging sequence similarity to make predictions. More details on model architecture, data preprocessing, train-test split generation, and training are provided in the Methods section.

We evaluated training convergence by tracking perplexity on a validation dataset held out from the training set (Figure 1E). Perplexity quantifies the model’s uncertainty in predicting codons, representing the average number of codon choices the model considers per amino acid. It is defined as the exponential of the cross-entropy loss, with lower values indicating more confident predictions with reduced uncertainty and fewer equally probable alternatives. During training, perplexity dropped from an initial value of 2.5 to below 1.5 after 600K examples, indicating that the model shifted from assigning probability broadly across many possible codons to focusing on a smaller set of preferred choices. This early decrease suggests that Trias quickly learned strong codon preferences. Although Trias was not explicitly provided with the codon table, it quickly learned to select valid synonymous codons for each amino acid (Supplementary Figure S1 and S2).

To analyze how Trias learns amino acid representations via its encoder module, we visualized the t-SNE projection of the final encoder hidden layer for 20 randomly selected human protein sequences (Figure 1F). The resulting representation shows clustering of amino acids based on their biochemical properties, with similar results to the ESM-1b model (Rives et al., 2021) that groups amino acids according to shared biochemical characteristics. Hydrophobic residues, for instance, form distinct groups separate from polar and charged amino acids. This suggests that Trias learns biologically meaningful representations that may contribute to improved codon selection. Supplementary Figure S3 presents the same projection using alternative labeling schemes, further highlighting distinct biochemical groupings.

To illustrate how Trias conditions codon predictions on protein context, we visualized cross-attention weights for an example gene COX7B2 (Figure 1G). Cross-attention indicates how much each predicted codon attends to different positions in the input protein sequence. Each row corresponds to a codon, and each column to an amino acid. The example shown represents a single attention head, averaged across all layers. Cross-attention maps for all heads are provided in Supplementary Figure S4. The strong diagonal signal indicates that codon choice is primarily influenced by the identity of its target amino acid. The vertical band at the start highlights the species tag, which influences codon selection across the sequence. Off-diagonal signals show that the model also attends to distant residues, suggesting that Trias incorporates both local and long-range context during codon generation.

### 2.2 Trias captures functional constraints on codon choice for sequence generation

**Figure 2:**
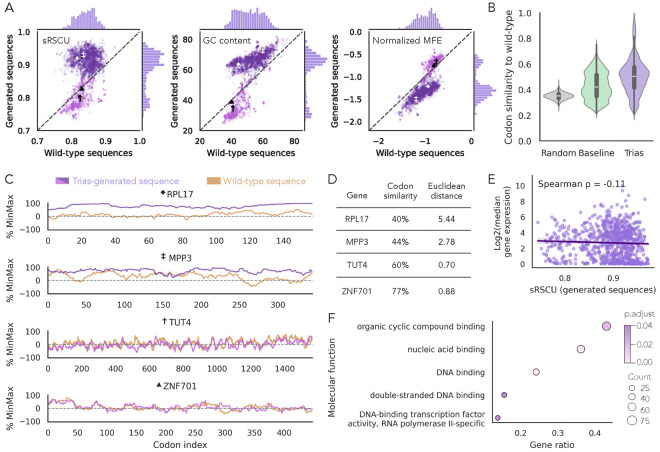
Sequence-level analysis of Trias-generated human codon sequences. (A) Comparison of Trias-generated sequences to their wild-type counterparts across scaled relative synonymous codon usage (sRSCU), GC content, and minimum free energy (MFE, normalized by sequence length) in the human test set (~6,000 sequences, including isoforms). Sequences were colored purple or pink according to high or low generated sRSCU values to facilitate comparison across metrics. (B) Codon similarity between Trias-generated sequences and wild-type sequences, benchmarked against a baseline model selecting the most frequent codon at each position and a model that randomly selects synonymous codons for every amino acid. (C) %MinMax profiles illustrate codon usage fluctuations across four genes with naturally low sRSCU (0.81–0.83). TUT4 and ZNF701 maintain low sRSCU similar to wild-type. RPL17 and MPP3 have a higher predicted sRSCU (~0.92). (D) Codon similarity and Euclidean distance between profiles (normalized by sequence length) quantify deviations from the wild-type codon usage profiles. (E) Log2-transformed median gene expression across all tissues in the GTEx dataset plotted against predicted sRSCU values for 810 human test set genes found in GTEx. (F) Gene ontology (GO) enrichment analysis of genes with predicted low sRSCU (≤0.86). Shown are significantly enriched GO terms (adjusted p-value < 0.05).

To evaluate the generative performance of Trias, we generated codon sequences for each protein in the human test set (~6,000 sequences, including multiple splice isoforms of some genes) using a greedy search decoding strategy. In this approach, the model selects the most probable codon at each step based on the previously generated sequence. We then compared these generated sequences to their wild-type counterparts by analyzing scaled relative synonymous codon usage (sRSCU), GC content, and minimum free energy (MFE) (Figure 2A). MFE, normalized by sequence length, was computed using the ViennaRNA package (Lorenz et al., 2011) to assess mRNA stability. sRSCU quantifies codon usage bias, where a value of 1 for a sequence indicates that the most frequent codon was chosen at each site. Higher sRSCU values reflect a preference for common codons, while lower values indicate the use of rare codons. See the Methods section for a detailed description of how sRSCU is calculated for a sequence.

Comparison of wild-type and Trias-generated sequences for each gene revealed a distinctive pattern (Figure 2A). The naturally occurring sequences had a unimodal distribution of sRSCU, but the generated sequences fell into two populations: the bulk of the sequences, spanning the full range of natural sRSCU values, were generated to have more optimal codons than their natural counterparts, but a distinct, smaller population of sequences were generated with low sRSCU that more closely matched, or even under-estimated, the low natural sRSCU of these sequences. GC content followed a similar bimodal pattern, and with a stronger overall correlation, as did MFE. Sequences with high sRSCU values, where the model overestimated codon usage, exhibited higher GC content and stability.

As GC content, mRNA structure, and codon choice are intertwined, with an intrinsic link between G-C basepairs and mRNA structure stability, we probed whether Trias learned fundamental preferences related to structure and mRNA stability. Although the overall correlation was weak, codons with higher sRSCU values tend to have higher GC content in humans (Supplementary Figure S5). The higher correlation between the GC content of wild-type and Trias-generated sequences compared to the correlation of sRSCU suggests that GC preferences might drive Trias’s codon preferences.

To establish baseline expectations, we compared Trias to two models: a random model that samples uniformly from the set of synonymous codons at each position and a frequency-based baseline model that always selects the most common codon for each amino acid. On average, sequences generated by Trias were 50% similar to their wild-type counterparts (Figure 2B), based on codon identity, compared to 44% for the frequency-based baseline model and 37% for the random model. The scatter plots of sRSCU, GC content, and MFE for those two other models did not show the bimodal distribution observed in Trias outputs (Supplementary Figure S6). These results indicate that Trias learns sequence-specific codon preferences beyond random selection and simple frequency-based patterns.

Overall, the model prioritizes frequently used codons and more structural stability for most genes, beyond their natural sequences, while in a subset of cases it preserves natural codon usage patterns that lead to lower stability. To further investigate the bimodal pattern of codon usage we observed in generated sequences, we selected four genes with naturally low sRSCU (0.81–0.83): two where Trias preserved rare codon usage (TUT4 and ZNF701), and two where it predicted more frequent codons than in the wild-type (RPL17 and MPP3). We plotted their true %MinMax profiles alongside those of their Trias-generated counterparts (Figure 2C). The %MinMax profile tracks relative codon usage fluctuations along a sequence, with low values indicating clusters of rare codons (Rodriguez et al., 2018). We further assessed codon similarity and used the Euclidean distance between codon usage profiles, normalized by sequence length, to quantify differences in codon distribution (Figure 2D). Generated sequences for TUT4 and ZNF701 maintained low sRSCU values, aligning with wild-type sequences, while RPL17 and MPP3 were predicted with higher sRSCU (~0.92) and deviated more strongly from the wild-type %MinMax profiles.

As ribosomal proteins such as RPL17 tend to be highly expressed, and expression has been linked to codon usage in some model organisms, we investigated whether Trias assigns higher sRSCU values based on gene expression levels. We used gene expression values (measured as mRNA abundance from RNA-seq) from GTEx, taking the median expression across all tissues for 810 genes in our human test set that matched GTEx entries. Interestingly, we found no significant correlation between predicted sRSCU and gene expression (Figure 2E). Similarly, natural sRSCU values also showed no correlation with gene expression (Supplementary Figure S7). These results suggest that Trias does not assign frequent codons based on gene expression and that highly expressed human genes do not necessarily favor frequent codons, in keeping with evolutionary analyses (Bénitière et al., 2025). As expected, we observed a weak correlation between codon similarity and wild-type sRSCU (Supplementary Figure S7). This reflects the model’s tendency to overestimate sRSCU, resulting in greater similarity for sequences that naturally have higher sRSCU values.

We showed that Trias preserves low sRSCU values in some genes, such as TUT4 and ZNF701, while predicting higher sRSCU for others. To investigate whether this pattern reflects functional constraints, we performed a gene ontology (GO) analysis on genes with predicted low sRSCU (≤0.86) (Figure 2F). The GO analysis revealed a significant enrichment for nucleic acid binding functions in these predicted low-sRSCU genes, suggesting that rare codon usage in these genes is not random but may be under selection to maintain functional roles. In contrast, genes predicted with high sRSCU showed no significant GO enrichment.

### 2.3 Sequence context influences rare codon predictions

**Figure 3:**
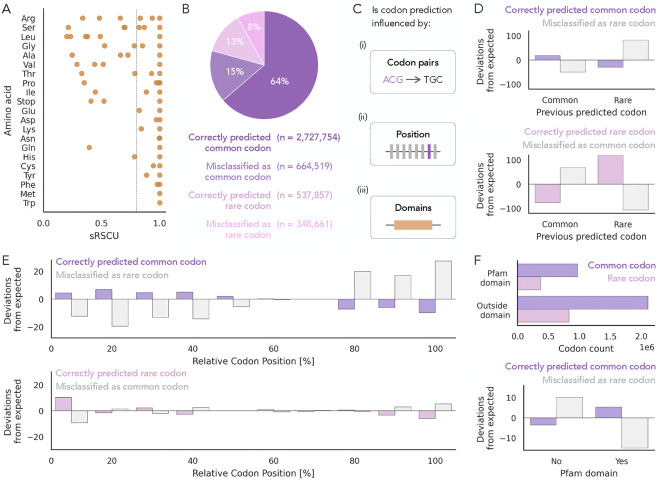
Contextual influences on codon prediction. (A) Rare codons were defined using an sRSCU threshold of <0.8. (B) Predictions for all 4,351,017 codons from ~6,000 human sequences in the test set were categorized into four classes based on whether the predicted codon was common or rare and whether it matched the wild-type codon. (C) To test whether codon predictions are influenced by previous codon identity, sequence position, or domain context, we performed separate chi-squared tests for true common codons (correctly predicted common and misclassified as rare) and true rare codons (correctly predicted rare and misclassified as common). For each analysis, observed prediction frequencies were compared to expected frequencies under the assumption of independence between prediction outcome and the feature. (D–F) Standardized residuals are plotted to show deviations from expected frequencies. (D) Prediction outcomes were significantly associated with the identity of the previous codon (p-value < 1e-5). The strongest deviations were observed for rare codons, which were more likely to follow rare codons than expected. (E) Prediction outcomes varied significantly with codon position (p-value < 1e-5). The largest deviations were observed at the 5^′^ end (first 40%) and 3^′^ end (last 30%) for true common codons, and within the first 10% for true rare codons. (F) Over 11,000 Pfam domains were identified using InterProScan across the test set proteins. Codon predictions were significantly affected for true common codons (p-value < 1e-5), but not for true rare codons.

To understand in what context Trias prefers rare codons over common codons, we performed a rare codon analysis. We first determined a threshold for rare codons based on the sRSCU value (Figure 3A). Codons with sRSCU<0.8 were classified as rare, while those with higher values were considered common codons. This allowed us to classify all 4,351,017 codon predictions from the around 6,000 human test set sequences into four categories based on their wild-type identity and model prediction: (i) correctly predicted common codon, (ii) misclassified as rare codon (common codons incorrectly predicted as rare), (iii) correctly predicted rare codon, and (iv) misclassified as common codon (rare codons incorrectly predicted as common) (Figure 3B).

To test whether certain sequence features influence the model’s codon predictions, we used chi-squared tests to compare the observed and expected frequencies of the four prediction outcome categories. Separate contingency tables were constructed for true common codons (correctly predicted common and misclassified as rare) and true rare codons (correctly predicted rare and misclassified as common), and chi-squared tests were performed to evaluate if a sequence feature affected the accuracy of the model on common or rare codons. Expected frequencies were calculated under the assumption that prediction outcome and the sequence feature being tested were independent. We tested if predictions were affected by the previous codon, the position in the sequence, and whether or not the codon was located within a protein domain (Figure 3C). Standardized residuals were used to highlight the individual contributions of each feature to the overall test statistic. Details on how expected values were calculated are provided in the Methods section, and observed frequencies for each category are reported in Supplementary Tables S1–S4.

First, we found that codon predictions were influenced by the identity of the previously predicted codon (chi-squared test, p-value<1e-5). Rare codons were more likely to follow other rare codons than expected (Figure 3D). A similar trend was observed for common codons, where common codon predictions more often followed other common codons, although the effect was less pronounced. Supplementary Figure S8 shows the full breakdown of these effects both by individual codons and aggregated by the preceding amino acid.

We then examined whether codon position within the sequence, measured across the relative length of each coding sequence, influences the model’s predictions (Figure 3E). The chi-squared tests indicated a significant association between codon position and prediction outcome (p-value < 1e-5). Common codons misclassified as rare were less frequent near the 5^′^ end (first 40%) and 3^′^ end (last 30%) of sequences. Correctly predicted rare codons were enriched within the first 10% at the 5^′^ end. These deviations contributed the most to the overall test statistic. This suggests that Trias tends to overestimate rare codon usage toward the end of coding regions, but more accurately predicts rare codons at the beginning.

Finally, we investigated whether codon predictions are affected by protein domain localization. Using InterProScan, we identified 11,000 Pfam domains across the test set and labeled codons as either within or outside of these domains. Around 30% of both rare and common codons were located within Pfam domains. We found that predictions were significantly affected by domain location for common codons, but not for rare codons (Figure 3F). Within domains, common codons were more often predicted as such, and misclassification of common codons as rare was strongly reduced. Outside of domains, common codons were more frequently misclassified as rare. These results suggest that codon usage within domains is more constrained, and Trias more closely aligns with wild-type codon choice for common codons in these regions.

### 2.4 Rare codon contexts bias generative models toward continued rare codon use

As shown in Figure 2A, Trias-generated sequences generally favor high-frequency codons even for genes whose natural sequence uses rare codons. However, a distinct subset of sequences are generated with rare codons, resulting in codon usage patterns that more closely resemble their wild-type counterparts. In fact, in these low-sRSCU genes, Trias tends to slightly overuse rare codons, leading to lower predicted sRSCU values than those of the wild-type sequences. This is consistent with our earlier observation (Figure 3D) that rare codons were more likely to be correctly predicted or misclassified as rare when the previous codon was also predicted to be rare. This likely reflects patterns observed in natural sequences, where rare codons are not randomly distributed but often occur in large clusters (Clarke and Clark, 2024).

**Figure 4:**
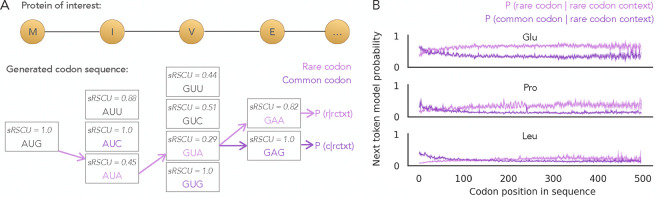
Effect of codon context on next token probabilities in Trias. (A) Next token probability is calculated for selecting the rarest (lowest sRSCU) or most common (highest sRSCU) codon, given that all preceding codons are rare. These are denoted as *P*(*r* | rctxt) and *P*(*c* | rctxt), with rctxt: rare codon context; r: rare codon; c: common codon. (B) Mean probabilities and confidence intervals across ~2,500 human sequences in the test set (sequence length ≤500 codons) are shown for three amino acids: glutamate (Glu), proline (Pro), and leucine (Leu).

To better understand how rare codon contexts influence subsequent predictions, we designed an *in silico* experiment where all preceding codons were constrained to be rare. Specifically, we analyzed around 2,500 human sequences in the test set with ≤500 codons and evaluated the model’s probability of selecting the next codon when all preceding codons were suboptimal, i.e., the lowest sRSCU per amino acid (Figure 4A). At each position, the next codon was constrained to be either the most frequent codon (highest sRSCU) or a rare codon (lowest sRSCU). We hypothesized that if rare codons were selected in previous steps, Trias would adapt to that context by assigning higher probabilities to subsequent rare codons throughout the sequence, even though globally such choices would be suboptimal. Consistent with our hypothesis, Trias favored rare codons in rare codon contexts, assigning them higher probabilities than frequent codons at the next position (Figure 4B). This trend was observed across all amino acids, with smaller probability differences for amino acids that have more synonymous codons (Supplementary Figure S9). We conclude that the model learned a form of internal consistency: although it is likely to predict preferred codons in most situations, it can detect genes that have stretches of rare codons and continue generating additional rare codons in these sequences. The subset of sequences that are generated to have low sRSCU may have distinctive features that the model has learned to associate with extended stretches of slow codons.

### 2.5 Trias generates codon sequences that align with experimental data

**Figure 5:**
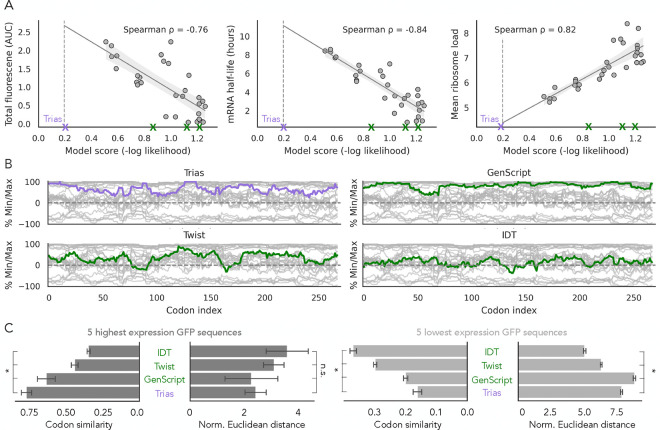
Trias-predicted codon sequences correlate with experimental data and differ from commercial codon optimization tools. (A) Correlation between Trias-predicted sequence likelihood and experimentally measured properties of 30 GFP codon variants expressed in HEK293 cells (data from Bicknell et al. (2024)). The purple cross marks the Trias-generated sequence obtained via greedy decoding (score = 0.19), with the regression line extrapolated up to this point. AUC: area under the curve quantifies total GFP fluorescence over time. (B) %MinMax codon usage profiles of all 30 GFP variants (gray) compared to sequences generated by Trias and three commercial codon optimization tools (GenScript, Twist, IDT). Trias likelihood scores for sequences generated by these tools were 0.86, 1.13, and 1.22 respectively (green markers in A). (C) Codon similarity and normalized Euclidean distances comparing Trias and commercial tool-generated sequences to the five highest- and lowest-expression GFP variants. Asterisks (*) denote statistical significance (p-value<0.05); error bars show standard deviation.

To evaluate the connection between Trias predictions and protein output, we benchmarked its predictions against experimental data from Bicknell et al. (2024) and commercially available codon optimization tools. The study from Bicknell et al. (2024) tested 30 different encodings of GFP, differing only in their codon composition, that were transfected into HEK293 cells as N1-methylpseudouridine-modified GFP mRNAs. The 30 variants were selected based on a grid of three distinct sRSCU and three different MFE values, with five variants chosen per region (Supplementary Figure S10). These mRNAs were assessed for GFP protein expression (measured as total fluorescence), mRNA half-life, and mean ribosome load. Experimental measurements were averaged across three biological replicates. We scored each sequence by computing the negative log-likelihood of the model’s probabilities for each codon sequence, normalized by sequence length, and tested correlations with experimental measurements (Figure 5A). The scores of our model showed strong correlations with GFP protein expression (*ρ* = −0.76), mRNA half-life (*ρ* = −0.84), and mean ribosome load (*ρ* = 0.82). Despite being trained only on natural sequences without any experimental data, Trias learns codon patterns that align with experimentally measured properties. Moreover, a simple calculation of sRSCU is insufficient as a predictor; high- and mid-sRSCU codon sequence variants show a wide range of expression levels (Supplementary Figure S10B). In contrast, our model captures additional sequence context beyond codon frequency and shows improved predictive power.

Interestingly, our model assigned relatively high likelihood to some low-sRSCU variants with low protein expression, such that the model score over-predicted protein output. This observation is consistent with our earlier finding that Trias tends to assign high likelihood to sequences with clusters of rare codons, even when such sequences are globally suboptimal. It supports the idea that Trias learns a sort of internal consistency, with higher scores for sequences that have similar codon usage throughout the gene. As natural sequences are not all under selective pressure for high expression, we posit that our model is accurately capturing attributes of natural patterns of codon usage and that these may not always align with the priorities for synthetic sequences. Nonetheless, the sequences with the highest likelihoods did produce the most protein.

In addition to scoring the 30 experimental GFP variants, we further generated a human GFP codon sequence using a greedy decoding approach from the Trias model. Its likelihood score exceeded the scores of all experimentally tested sequences. Since Trias generates sequences by selecting the most probable codon at each step, deviations from its predicted sequence receive lower scores. Given the strong correlation between model scores and experimental protein expression, we extrapolated the regression line in Figure 5A to estimate a potential expression level of around 2.67 for this Trias-optimized sequence.

We also compared this optimal Trias-generated sequence to those produced by commercially available codon optimization tools, such as GenScript, Twist, and IDT. Figure 5B shows %MinMax profiles of all generated sequences together with the 30 GFP variants (gray lines). Trias- and GenScript-generated sequences predominantly follow the upper range of the %MinMax profile, indicating a preference for more frequently used codons. Following the upper range entirely would correspond to selecting the most frequent codon at every position, an approach more closely reflected by GenScript than by Trias. Trias also prefers frequent codons, but less consistently than GenScript and more than Twist or IDT. However, despite their similar profiles, Trias ranked the GenScript sequence significantly lower, with a score of 0.86, and Twist and IDT scored even lower, with scores of 1.13 and 1.22, respectively (Figure 5A).

To further quantify these observations, we compared codon sequences produced by Trias and commercial codon optimization tools to the five highest- and five lowest-expressing GFP variants using codon similarity and Euclidean distance (normalized by sequence length) of the %MinMax profiles (Figure 5C). The Trias-generated sequence showed significantly higher codon similarity to the five highest-expressing variants (76%) than to the lowest-expressing ones (16%), a trend also observed for GenScript (63% vs. 20%). In contrast, Twist and IDT sequences showed smaller differences between high- and low-expression variants, with codon similarities ranging between 30% and 44%. A similar trend was observed for the Euclidean distances, where the %MinMax profiles of Trias and GenScript sequences more closely resembled those of the highest-expressing variants and diverged more from the lowest-expressing ones. Again, Twist and IDT exhibited smaller differences, aligning more closely with mid-sRSCU GFP variants (Supplementary Figure S11). Taken together, these results suggest that Trias-generated sequences more effectively capture codon usage patterns linked to high protein expression compared to commercially optimized sequences.

## 3 Discussion

Trias is a species-aware encoder-decoder model trained on 10 million vertebrate coding sequences to reverse translate protein sequences into codon sequences. Through its generative training, Trias accurately narrows its predictions from all 64 codons to the correct synonymous codons for each amino acid, without being explicitly provided with the codon table or any hard-coded constraints. Trias learns codon usage directly from natural sequences, capturing biologically meaningful patterns beyond simple frequency-based approaches. This data-driven strategy presents a promising alternative for designing codon-optimized mRNA sequences.

Our analysis reveals that Trias generally favors common codons, beyond their frequency in natural sequences, and generates sequences with higher GC content and increased mRNA stability. However, it correctly predicts high use of rare codons in specific genes, suggesting that it has learned functional constraints on codon choice. Notably, gene ontology analysis showed that these genes are enriched for nucleic acid-binding functions. Beyond an overall preference for rare or common codons in different genes, Trias also learns position-dependent preferences for rare codons. Rare codons were more frequently predicted at the 5^′^ end of coding sequences, and within annotated protein domains, common codons were misclassified as rare less often. These observations indicate that Trias captures codon usage preferences shaped by context, going beyond frequency alone.

An implicit assumption in many applications of nucleotide foundation models for sequence design is that higher-likelihood sequences will correspond to higher protein expression. While this may hold in some cases, natural mRNAs are not necessarily optimized for high expression. Despite this conceptual limitation and without explicit training on experimental protein expression data, Trias’ predicted scores for GFP codon variants show strong correlations with protein output, mRNA half-life, and ribosome load. Thus, our results validate the approach of learning from natural optimization in designing synthetic mRNAs for high expression.

The exceptions to this pattern are important to consider. We find that Trias’ autoregressive decoding introduces a form of local internal consistency, where rare codon choices are more likely to be followed by additional rare codons. This likely reflects patterns in natural sequences, where rare codons tend to cluster (Clarke and Clark, 2024). However, this behavior can lead to generation of sequences with codon usage that may echo nature but does not correspond to high expression. It may also lead to biased overestimation of rare codon usage. These findings have broader implications for the use of generative language models in predictive settings. Off-the-shelf models trained on natural sequences may carry biases that affect downstream applications, not only for zero-shot prediction of protein expression, but also for tasks such as variant effect prediction, where sequence likelihoods are often used as proxies for functional impact or pathogenicity. For Trias, fine-tuning on protein expression data could help reduce these biases and further enhance its ability to design codon sequences optimized for high protein expression.

Foundation models of nucleic acid sequence have become promising tools for synthetic mRNA sequence design, with broad applications in recombinant protein production and mRNA-based therapeutics. Unlike commercial codon optimization tools that rely on predefined rules, Trias leverages a data-driven approach that generalizes across species and functional contexts. Our benchmarking demonstrated that Trias-generated sequences more closely resemble high-expression GFP variants than those from commercial tools. In contrast to other deep learning models, Trias focuses on vertebrate sequences to gain specific insight into the rules most likely to apply to human mRNA design; these rules can differ in key ways from prokaryotic translation constraints. Despite training on a smaller, phylogenetically narrower dataset, our model achieves strong performance, supporting the value of models tailored to specific biological scenarios. Trias is distinct in integrating generative sequence design into its architecture, limiting our ability to compare it directly to published codon language models that focus on predictive tasks rather than sequence generation. Nonetheless, our benchmarking against experimental protein expression data demonstrates that Trias effectively captures biologically relevant codon usage patterns that translate to improved protein output.

Ultimately, a complete model for synthetic mRNA design must incorporate regulatory features beyond the codon sequence itself. Untranslated regions (UTRs) play key roles in mRNA stability and translation efficiency (Mayr, 2017; Leppek et al., 2018), and the UTR sequence can affect the impact of codon choice (Lyons et al., 2024). Understanding these interactions could further refine the model’s ability to design synthetic mRNA sequences; recent work has shown that incorporating UTRs can improve predictions of mRNA properties and protein expression (Li et al., 2025). A model of synthetic mRNA must also consider the impact of chemical modifications such as with N1-methylpseudouridine that are necessary to reduce innate immune reactions to therapeutic mRNAs (Metkar et al., 2024). These modifications, not found in natural mRNAs, may affect mRNA structure and translation efficiency. Therefore, fine-tuning Trias on chemically modified sequences will be important to account for these effects and adapt codon choices accordingly. Future work to expand the scope of our model can fully unlock the potential of generative models for mRNA design in synthetic biology and therapeutic applications.

## 4 Methods

### 4.1 Datasets

#### 4.1.1 Training and test dataset

We downloaded all vertebrate sequences from the NCBI RefSeq database^4^ on August 5th, 2024. To ensure data quality, we applied the following filtering criteria: sequences labeled as “pseudo” were excluded, and only records containing the feature tags “CDS” and “translation” were kept. We verified that each sequence was complete, initiated with the start codon “ATG” and terminated with one of the three stop codons (“TAA” “TGA” or “TAG”). Additionally, the translated protein sequences were required to start with methionine “M” and end with a stop symbol “*”.

After processing the downloaded records, we filter the mRNA sequences according to the length of the coding region. This step resulted in approximately 26 million sequences. To reduce redundancy, we clustered sequences with 90% or greater nucleotide similarity using MMSeqs2 and kept only one representative sequence per cluster. This step reduced the data set to around 10.2 million sequences.

To evaluate model performance, we created a train-test split from our curated dataset of 10.2 million coding sequences. For the test set, we randomly selected 300 human genes from each expression category (high, medium, and low) based on GTEx expression levels, ensuring a diverse range of expression profiles. These 900 genes were translated into protein sequences and used to query the rest of the dataset using BLAST. Any sequences in the remaining dataset with significant protein similarity to these test genes were removed and added to the test set to avoid overlap. This resulted in a test set of approximately 200,000 sequences, including the original 900 human genes. In total, the test set contains approximately 6,000 human coding sequences, including multiple splice isoforms of some genes. The final training set consisted of all 10 million remaining sequences from the curated dataset after removing those with significant protein similarity to the test genes. This approach ensured that the test set, used for performance evaluation, did not include sequences with close homologues in the training set. To monitor training progress, we randomly sampled 1% of the training data as a validation set, with sampling balanced across species.

#### 4.1.2 Gene expression data from GTEx

The GTEx dataset provides gene expression data across 54 human tissues from approximately 1,000 individuals, measured via bulk RNA sequencing (RNA-seq). For this study, gene expression data were obtained from the GTEx Portal^5^ on December 11th 2024, from the GTEx_Analysis_2017-06-05_v8_RNASeQCv1.1.9_gene_median_tpm.gct.gz file, which contains median gene-level TPM values per tissue. Genes with no expression (TPM = 0) in any tissue were excluded. We then computed the median TPM across all tissues and retained only genes that matched those in our human test set.

### 4.2 Model architecture and training

Trias is based on the HuggingFace implementation of the BART encoder-decoder architecture (Lewis et al., 2019). The input sequence length is set to 2,048 tokens, with the first token reserved for a species tag to ensure species-specific optimization. The vocabulary consists of 64 codon tokens, 20 amino acid tokens, 640 species-specific tokens, and 4 special tokens (</s>, <unk>, <pad>, <mask>).

The model uses the BartForConditionalGeneration class, with 15% of amino acid tokens in the protein sequence masked during training. Flash attention is applied to improve memory efficiency and computational speed. Optimization is performed using the Adam optimizer with a cosine learning rate schedule and warm-up steps. The learning rate starts at 1e-4 and a weight decay of 0.01 is applied. Dropout is set to 0.1. The model is trained for 650,000 steps, corresponding to approximately two epochs.

Training is carried out on an NVIDIA A100 GPU with 80 GB of memory. The batch size is set to 8, and gradient accumulation is used with an accumulation factor of 4, resulting in an effective batch size of 32. The architecture consists of 6 encoder layers and 6 decoder layers, each with 8 attention heads. The hidden size of the feedforward networks is 2,048 for both the encoder and decoder. This configuration results in a model with approximately 47M trainable parameters.

### 4.3 Data analysis and performance metrics

#### 4.3.1 Gene ontology enrichment analysis

To investigate functional constraints on codon usage, we performed a gene ontology (GO) enrichment analysis on genes with predicted low and high sRSCU values. Prior to analysis, we removed isoforms from the human test set, resulting in a non-redundant set of 879 genes (down from 6,165 sequences, including isoforms). For each gene, we aggregated predicted and natural sRSCU values by taking the mean across isoforms. We classified genes into two groups based on their predicted sRSCU: genes with low sRSCU (≤0.86) and high sRSCU (>0.86). We then performed GO analysis to identify functional terms overrepresented in these subsets compared to the full set of 879 human test genes. GO term enrichment was assessed using the gprofiler Python package, with the background set defined as all 879 genes in the test set. We applied Bonferroni correction for multiple hypothesis testing and set a significance threshold of p-value < 0.05. GO terms related to molecular function, biological process, and cellular component categories were considered in the analysis.

#### 4.3.2 Codon usage calculation

To quantify codon usage bias, we computed the scaled relative synonymous codon usage (sRSCU) for every codon in each species based on species-specific codon frequency in the training set. sRSCU normalizes codon frequency within synonymous codon categories, allowing direct comparisons across different amino acids. It measures how frequently a given codon is used relative to the expected uniform distribution of synonymous codons for an amino acid. The relative synonymous codon usage (RSCU) for a given codon c is calculated following Sharp et al. (1986):

(1) $$RSCU_{i,j}=\frac{c_{i,j}}{\frac{1}{n_{i}}\sum_{j=1}^{n_{i}}c_{i,j}},$$

where $c_{i,j}$ is the observed frequency of the jth codon for the ith amino acid in the training set for a specific species, and $n_{i}$ is the number of synonymous codons for the ith amino acid. To derive sRSCU, we scale RSCU values by dividing by the highest RSCU value for each amino acid within a species:

(2) $$sRSCU_{i,j}=\frac{RSCU_{i,j}}{max\left(RSCU_{i,1},\;RSCU_{i,2},\;\ldots ,\;RSCU_{i,n_{i}}\right)}$$

This ensures that the most frequently used synonymous codon receives a score of 1, while other synonymous codons are scaled accordingly. To calculate the sRSCU for a gene, we computed the average sRSCU value of all codons within that gene.

#### 4.3.3 Expected value calculation for chi-squared tests

We used chi-squared tests to assess whether prediction outcomes were associated with specific sequence features (previous codon, codon position, and domain localization). Codon predictions from the test set were categorized into four classes (correctly predicted common, misclassified as rare, correctly predicted rare, or misclassified as common). Observed frequencies for all four prediction categories across each sequence feature are reported in Supplementary Tables S1–S4. For the chi-squared tests, we constructed separate contingency tables for true common codons (correctly predicted common and misclassified as rare) and true rare codons (correctly predicted rare and misclassified as common). Expected counts were calculated under the null hypothesis that codon prediction categories are independent of the tested feature. Expected values $E_{ij}$ were computed as follows:

(3) $$E_{ij}=\frac{R_{i}\;\cdot \;C_{j}}{N},$$

where $R_{i}$ is the total count for feature level i, $C_{j}$ is the total count for prediction category j, and N is the total number of codon predictions. To identify which feature levels contributed most to significant associations, we also computed standardized residuals.

#### 4.3.4 Pfam domain annotation

We used InterProScan to identify protein domains in our test set. It scans protein sequences against a comprehensive collection of databases to annotate known domains, families, and functional sites. InterProScan was run locally on all ~6,000 human protein sequences in the test set. From the resulting annotations, we extracted Pfam domain matches and kept only those with e-values <1e-5 to ensure high-confidence predictions. The e-value indicates the number of matches expected by chance, with lower values reflecting higher significance. Codons were labeled as within a domain if they overlapped a detected Pfam domain, and as outside a domain otherwise.

#### 4.3.5 Codon usage profiles

To analyze codon usage variability along sequences, we computed %MinMax profiles, which quantify deviations in codon usage relative to expected frequency distributions. This approach is adapted from Rodriguez et al. (2018) and provides a measure of codon bias by comparing observed codon frequencies within a sliding window (size = 18) to the expected frequency range. Positive %MinMax values indicate a preference for frequently used codons, while negative values reflect increased usage of rare codons.

To compare codon usage patterns between sequences, we used normalized Euclidean distance between their %MinMax profiles. The Euclidean distance was computed across the profile and normalized by sequence length. This approach provides a quantitative measure of similarity in codon usage fluctuations along the sequence.

#### 4.3.6 Sequence likelihood scoring

To assess the likelihood of a codon sequence S, we define a scoring function L(S) based on the negative log-likelihood assigned by the model. Trias decodes codon sequences autoregressively, generating codons one at a time, where each codon is predicted based on the previously generated codons and the input protein sequence. The likelihood of a sequence $S=\left(c_{1},c_{2},\;\ldots ,c_{N}\right)$ is given by:

(4) $$L\left(S\right)=-\frac{1}{N}\sum_{i=1}^{N}logP\left(c_{i}|c_{1},c_{2},\;\ldots ,c_{i-1}\right),$$

where $P\left(c_{i}|c_{1},c_{2},\;\ldots ,c_{i-1}\right)$ is the model’s predicted probability for codon $c_{i}$, given the preceding sequence $\left(c_{1},c_{2},\;\ldots ,c_{i-1}\right)$. The log-likelihood is normalized by sequence length N. A lower L(S) value indicates a sequence that is more likely under the model, meaning the model assigns high probabilities to codon choices at each position. A score of 0 corresponds to a sequence where the model is fully confident in every prediction $\left(P\left(c_{i}\right)=1\right)$, while higher scores reflect more uncertain predictions. Trias uses greedy decoding to generate codon sequences, a decoding strategy in which at each step the model selects the most probable codon. Greedy decoding ensures high-likelihood outputs.

## Supplementary Material

Supplement 1

## Footnotes

1
https://www.genscript.com/gensmart-free-gene-codon-optimization.html

2
https://www.twistbioscience.com/resources/digital-tools/codon-optimization-tool

3
https://www.idtdna.com/pages/tools/codon-optimization-tool

4
https://ftp.ncbi.nlm.nih.gov/refseq/release/

5
https://www.gtexportal.org/home/
