# A generative language model decodes contextual constraints on codon choice for mRNA design

**DOI:** 10.1101/2025.05.13.653614

**Published:** 2025-06-06

**Authors:** Marjan Faizi, Helen Sakharova, Liana F. Lareau

**Affiliations:** 1 California Institute for Quantitative Biosciences, University of California, Berkeley, CA 94720, USA; 2 Center for Computational Biology, University of California, Berkeley, CA 94720, USA; 3 Department of Bioengineering, University of California, Berkeley, CA 94720, USA; 4 Chan Zuckerberg Biohub, San Francisco, CA 94158, USA

## Abstract

The genetic code allows multiple synonymous codons to encode the same amino acid, creating a vast sequence space for protein-coding regions. Codon choice can impact mRNA function and protein output, a consideration newly relevant with advances in mRNA technology. Genomes preferentially use some codons, but simple optimization methods that select preferred codons miss complex contextual patterns. We present Trias, an encoder-decoder language model trained on millions of eukaryotic coding sequences. Trias learns codon usage rules directly from sequence data, integrating local and global dependencies to generate species-specific codon sequences that align with biological constraints. Without explicit training on protein expression, Trias generates sequences and scores that correlate strongly with experimental measurements of mRNA stability, ribosome load, and protein output. The model outperforms commercial codon optimization tools in generating sequences resembling high-expression codon sequence variants. By modeling codon usage in context, Trias offers a data-driven framework for synthetic mRNA design and for understanding the molecular and evolutionary principles behind codon choice.

## 1 Introduction

Synthetic messenger RNA technology has emerged as a transformative tool for biotechnology, opening new frontiers in synthetic biology, genome engineering, and medicine. mRNAs encoding proteins of interest can be delivered into cells for translation by the cell’s own machinery. A key challenge is to design mRNA sequences that drive high levels of heterologous protein expression. Natural mRNAs are shaped by overlapping constraints: coding sequences must ensure accurate protein synthesis, while the coding region and untranslated regions (UTRs) also control aspects of mRNA fate like translation initiation rate, structural stability, and half-life. All of these aspects of post-transcriptional regulation are subject to optimization for synthetic mRNA design (Sample et al., 2019; Lewis et al., 2024; Leppek et al., 2022; Zhang et al., 2023).

Design of the coding sequence presents a particular challenge. While the desired protein sequence may be pre-determined, there are many ways to encode the same protein sequence in mRNA. Most amino acids can be specified by two or more synonymous codons, so codon choice becomes a combinatorially complex problem. For example, the SARS-CoV-2 spike protein has 1,273 amino acids and can be encoded by more than 10^632^ different codon sequences (Kim et al., 2024). Within this vast range of possibilities, codon choice is not random; organisms favor certain codons, and these preferences vary by species. Yet, the underlying rules guiding optimal codon selection remain poorly understood (Wu and Bazzini, 2023).

Codon choice has subtle but powerful impacts on the outcomes of translation. Common codons are decoded faster and more accurately due to more abundant tRNAs, while rare codons slow translation (Liu, 2020). Slow translation can destabilize mRNAs through codon optimality mediated decay (Presnyak et al., 2015; Bazzini et al., 2016; Narula et al., 2019). It can also reduce the number of proteins made per mRNA; codon choice alone can create an eight-fold range in protein output (Tunney et al., 2018; Barrington et al., 2023; Lyons et al., 2024). However, fast translation is not always ideal. Multi-domain proteins can require precisely timed translation for proper folding and function (Kim et al., 2015; Yu et al., 2015; Kimchi-Sarfaty et al., 2007; Kirchner et al., 2017). Decoding the rules for codon choice thus has important applications for biotechnology and therapeutics (Lin et al., 2023a).

These observations underscore the need for sophisticated approaches to sequence design that consider biological context. The classic approach to codon optimization considers species-specific codon frequencies as well as mRNA structure and GC content. Most commercial tools rely on manually defined features and cannot fully capture the dependencies between codons. In parallel to these feature-based methods, some deep learning approaches learn directly from experimental data on ribosome distribution (Tunney et al., 2018; Jain et al., 2023). These models represent a major step forward by learning codon patterns from functional measurements, but they are based on hard to acquire data and may not capture complex sequence dependencies.

Recent advances in machine learning, particularly large language models (LLMs), have introduced powerful data-driven approaches that capture complex information from biological sequences. These models learn the underlying distributions and patterns from millions of naturally occurring sequences (Simon et al., 2024). LLMs can serve as foundation models that generalize across a wide range of biological tasks, either in a zero-shot setting (without fine-tuning or task-specific examples) or after fine-tuning. For example, the Evolutionary Scale Model (ESM), trained on millions of protein sequences, was fine-tuned to predict protein structure elements and mutation effects (Rives et al., 2021; Lin et al., 2023b). Similarly, genomic foundation models have been trained on millions of DNA sequences and fine-tuned for tasks ranging from variant effect prediction to the generation of novel CRISPR-Cas systems (Benegas et al., 2023; Karollus et al., 2024; Nguyen et al., 2024a,b; Brixi et al., 2025).

Building on the success of these large language models, several codon language models have recently been developed and trained on large datasets of mRNA coding sequences (Li et al., 2024; Constant et al., 2023; Outeiral and Deane, 2024; Sidi et al., 2024; Fallahpour et al., 2025; Naghipourfar et al., 2024). These data-driven models have the potential to uncover complex codon usage rules that traditional methods may not capture, providing an improved tool for codon optimization and a framework for studying synonymous codon choice. However, their use for synthetic mRNA design raises a fundamental question, as these models may not align perfectly with the optimization tasks to which they are applied. Natural mRNA sequences are optimized by evolution for their functional need, not necessarily for maximal expression. Understanding what these models learn about natural codon usage and how this knowledge translates to synthetic applications is critical for their successful use in sequence design.

While codon language models show promise, most existing models are based on encoder-only architectures that do not directly generate sequences. For example, CodonBERT (Li et al., 2024) and CO-BERTa (Constant et al., 2023) are fine-tuned to estimate sequence-level properties such as mRNA half-life or protein expression to support the design of synthetic sequences. These encoder-only models are well suited for classification and regression tasks, while encoder-decoder and decoder-only models are autoregressive architectures designed for sequence generation. Encoder-decoder models first map the input sequence into a latent representation, which is then decoded token by token into the target output (Cho et al., 2014). Decoder-only models use the input as a prompt and generate tokens autoregressively without an intermediate representation. Some codon models have explored generative capabilities. Constant et al. (2023) introduced an encoder-decoder model to reverse-translate protein sequences into codons for *E. coli*, and Sidi et al. (2024) trained an encoder-decoder model on short fragments from two bacterial and two yeast genomes, limiting the model’s ability to capture long-range dependencies across full-length coding sequences. Naghipourfar et al. (2024) presented EnCodon (encoder-only) and DeCodon (decoder-only) for prediction of synonymous variant effects and mRNA properties, but neither model was designed to generate codon sequences for a given protein.

Here, we present Trias, a generative language model based on an encoder-decoder architecture, trained on the full-length coding regions of 10 million vertebrate mRNA sequences for the task of reverse-translating protein sequences into codon sequences. Trias is distinct in focusing on eukaryotic sequences, capturing their distinct post-transcriptional sequence constraints to enable sequence design for expression in human cells. We show that Trias captures codon usage patterns that align with biological constraints by learning to generate coding sequences one codon at a time. The model favors frequent codons while preserving rare codons in functionally relevant contexts. With no training on experimental data, Trias predicted scores for synonymous variants of GFP that correlated strongly with measurements of their protein expression, ribosome load, and mRNA half-life in human cells.

Together, these findings highlight the potential of Trias as a data-driven method for codon optimization in recombinant protein production and mRNA-based therapeutics, as well as a tool for studying how codon usage is shaped by sequence context. To facilitate its broad application, we have released Trias on Hugging Face, where it enables reverse translation of protein sequences up to 2,046 amino acids in length into codon sequences for over 600 vertebrate species.

## 2 Results

### 2.1 Trias learns codon preferences and amino acid properties through generative training

**Figure 1:**
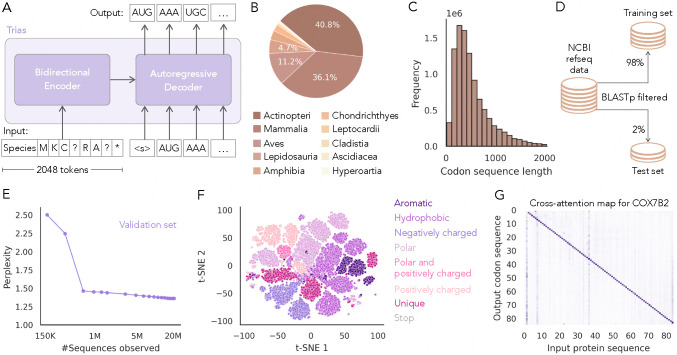
Model overview, dataset composition and training performance. (A) Trias is an encoder-decoder model that reverse translates protein sequences into codon sequences while incorporating species-specific codon preferences. During training, 15% of input tokens are randomly masked (indicated as ‘?’). (B) The model was trained on 10M codon sequences from 640 vertebrate species. (C) Distribution of codon sequence lengths in the dataset. (D) An initial test set was chosen at random and sequences with protein similarity to the initial test set were moved from the training to test set. To monitor training, we additionally randomly sampled 1% of the training data as a validation set. (E) Perplexity during training, showing a decrease from 2.5 to below 1.5 on the validation set. (F) Projection of the final encoder embedding layer for 20 randomly selected human protein sequences, with each dot representing an amino acid. (G) Cross-attention map for example gene, showing how each codon attends to the input amino acid sequence and the species tag. Each row corresponds to a codon, and each column to an amino acid. The example shown represents a single attention head, averaged across all layers.

Trias is a sequence-to-sequence model designed to reverse translate protein sequences into codon sequences (Figure 1A). It is based on the BART architecture with bidirectional encoder and autoregressive decoder modules, commonly used in natural language processing tasks such as machine translation (Lewis et al., 2019). The bidirectional encoder module of Trias processes the entire input protein sequence at once, capturing long-range dependencies between amino acids. It produces a latent representation of the sequence that is passed to the decoder. The autoregressive decoder then generates the corresponding codon sequence token by token, conditioning each predicted codon on the previously generated codons and the encoder’s representation. The model is trained by providing the encoder module with a corrupted version of each protein sequence with 15% of amino acid positions masked and then calculating the reconstruction loss of the generated codon sequence against the true codon sequence. To allow the model to learn species-specific preferences for codon usage, we prepend species tags to the protein input sequence.

Trias has 47 million parameters. It was trained on 10 million sequences from 640 vertebrate species sourced from the NCBI RefSeq database (Figure 1B). Sequences can be up to 2,046 tokens long (Figure 1C), exceeding the input limits of previous models. To ensure robust evaluation and prevent information leakage between the training and test sets, we removed homologous sequences based on protein-level similarity (Figure 1D); highly similar sequences between sets could allow the model to simply leverage sequence similarity rather than learning generalizable codon preferences.

We evaluated training convergence by tracking perplexity on a validation dataset held out from the training set (Figure 1E). Perplexity quantifies the model’s uncertainty in predicting codons, representing the average number of codon choices the model considers per amino acid. Lower values indicate more confident predictions with reduced uncertainty and fewer equally probable alternatives. During training, perplexity dropped from 2.5 to below 1.5 after 600K examples, indicating that the model quickly learned synonymous codon preferences. We also confirmed that, although Trias was not explicitly provided with the codon table, it quickly learned to select valid synonymous codons for each amino acid (Supplementary Figure S1 and S2).

We confirmed that the encoder module learned meaningful latent representations of amino acids by visualizing the t-SNE projection of the final encoder hidden layer for 20 randomly selected human protein sequences (Figure 1F, Supplementary Figure S3). Amino acids clustered by biochemical properties, similar to ESM-1b (Rives et al., 2021), with distinct groups for hydrophobic, polar, and charged residues. Next, to illustrate how Trias conditions its codon predictions on protein context, we visualized cross-attention weights for an example gene, COX7B2 (Figure 1G, Supplementary Figure S4). Cross-attention indicates how much each predicted codon attends to different positions in the input protein sequence. The strong diagonal signal indicates that codon choice is primarily influenced by the identity of its target amino acid. The vertical band at the start highlights the species tag, which influences codon selection across the sequence. Off-diagonal signals show that the model also attends to distant residues, suggesting that Trias incorporates both local and long-range context during codon generation.

### 2.2 Trias captures functional constraints on codon choice for sequence generation

**Figure 2:**
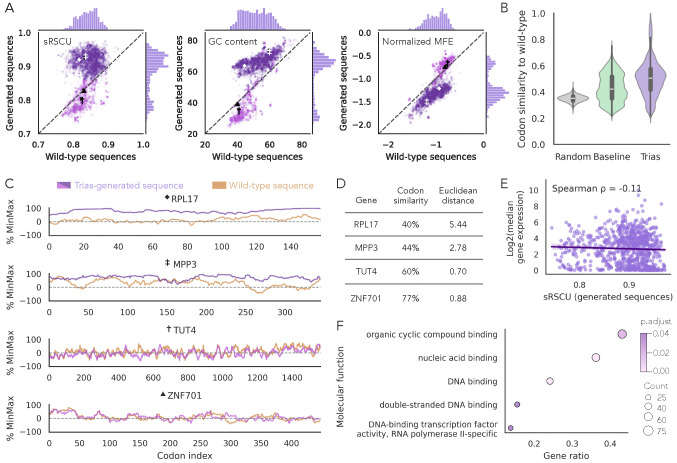
Sequence-level analysis of Trias-generated human codon sequences. (A) Comparison of Trias-generated sequences to their wild-type counterparts across scaled relative synonymous codon usage (sRSCU), GC content, and minimum free energy (MFE, normalized by sequence length, computed using the ViennaRNA package (Lorenz et al., 2011)) in the human test set (~6,000 sequences, including isoforms). Sequences were colored purple or pink according to high or low generated sRSCU values to facilitate comparison across metrics. (B) Codon similarity between Trias-generated sequences and wild-type sequences, benchmarked against a baseline model selecting the most frequent codon at each position and a model that randomly selects synonymous codons for every amino acid. (C) %MinMax profiles illustrate codon usage fluctuations across four genes with naturally low sRSCU (0.81–0.83), marked with symbols in panel A. TUT4 and ZNF701 maintain low sRSCU similar to wild-type. RPL17 and MPP3 have a higher predicted sRSCU (~0.92). (D) Codon similarity and Euclidean distance between profiles (normalized by sequence length) quantify deviations from the wild-type codon usage profiles. (E) Log2-transformed median gene expression across all tissues in the GTEx dataset plotted against predicted sRSCU values for 810 human test set genes found in GTEx. (F) Gene ontology (GO) enrichment analysis of genes with predicted low sRSCU (≤0.86). Shown are significantly enriched GO terms (adjusted p-value < 0.05).

To evaluate the generative performance of Trias, we generated codon sequences for each protein in the human test set (~6,000 sequences, including multiple splice isoforms of some genes) using a greedy search decoding strategy that selects the most probable codon at each step based on the previously generated sequence. We then compared these generated sequences to their wild-type counterparts by analyzing codon usage (measured as scaled relative synonymous codon usage, sRSCU, where a maximum value of 1 indicates that a sequence always uses the most common codons), GC content, and mRNA structure (measured as minimum free energy, MFE) (Figure 2A).

Comparison of wild-type and Trias-generated sequences for each gene revealed a distinctive pattern (Figure 2A). The naturally occurring sequences had a unimodal distribution of sRSCU, but the generated sequences fell into two populations. The bulk of the sequences, spanning the full range of natural sRSCU values, were generated to have more optimal codons than their natural counterparts, but a distinct, smaller population of sequences were generated with low sRSCU that more closely matched, or even under-estimated, the low natural sRSCU of these sequences. GC content followed a similar bimodal pattern, and with a stronger overall correlation, as did MFE. Sequences with high sRSCU values, where the model overestimated codon usage, exhibited higher GC content and stability.

As GC content, mRNA structure, and codon choice are intertwined, with an intrinsic link between G-C basepairs and mRNA structure stability, we probed whether Trias learned fundamental preferences related to structure and mRNA stability. Although the overall correlation was weak, codons with higher sRSCU values tend to have higher GC content in humans (Supplementary Figure S5). The higher correlation between the GC content of wild-type and Trias-generated sequences compared to the correlation of sRSCU suggests that GC preferences might drive Trias’ codon preferences.

To establish baseline expectations, we compared Trias to two models: a random model that samples uniformly from the set of synonymous codons at each position and a frequency-based baseline model that always selects the most common codon for each amino acid. On average, sequences generated by Trias were 50% similar to their wild-type counterparts (Figure 2B), based on codon identity, compared to 44% for the frequency-based baseline model and 37% for the random model. These baseline models did not show the bimodal distribution of sRSCU, GC content, and MFE observed in Trias outputs (Supplementary Figure S6). These results indicate that Trias learns sequence-specific codon preferences beyond random selection and simple frequency-based patterns.

We then explored possible explanations for the bimodal outcomes. Examples of the two classes are shown in Figure 2C. We selected four genes with naturally low sRSCU (0.81–0.83): two where Trias preserved rare codon usage (TUT4 and ZNF701), and two where it predicted more frequent codons than in the wild-type (RPL17 and MPP3). We plotted %MinMax profiles, showing relative codon usage fluctuations along a sequence, for the real and generated sequences, and assessed codon-level similarity and Euclidean distance between their codon usage profiles (Figure 2C,D). Generated sequences for TUT4 and ZNF701 closely matched the profiles of their wild-type sequences, while RPL17 and MPP3 deviated more strongly.

As ribosomal proteins such as RPL17 tend to be highly expressed, and expression has been linked to codon usage in some model organisms, we investigated whether Trias implicitly learns which genes are highly expressed and assigns them higher sRSCU values. We used gene expression values (measured as mRNA abundance from RNA-seq) from GTEx, taking the median expression across all tissues for 810 genes in our human test set that matched GTEx entries. Interestingly, we found no significant correlation between predicted or natural sRSCU and gene expression (Figure 2E, Supplementary Figure S7). These results suggest that highly expressed human genes do not necessarily favor frequent codons, in keeping with evolutionary analyses (Bénitière et al., 2025).

Next, to investigate whether the bimodal outcomes reflect functional constraints, we performed a gene ontology (GO) analysis on the subset of genes with predicted low sRSCU (≤0.86) (Figure 2F). The GO analysis revealed a significant enrichment for transcription factor genes in these predicted low-sRSCU genes, suggesting that rare codon usage in these genes may be under selection to maintain functional roles.

### 2.3 Sequence context influences rare codon predictions

**Figure 3:**
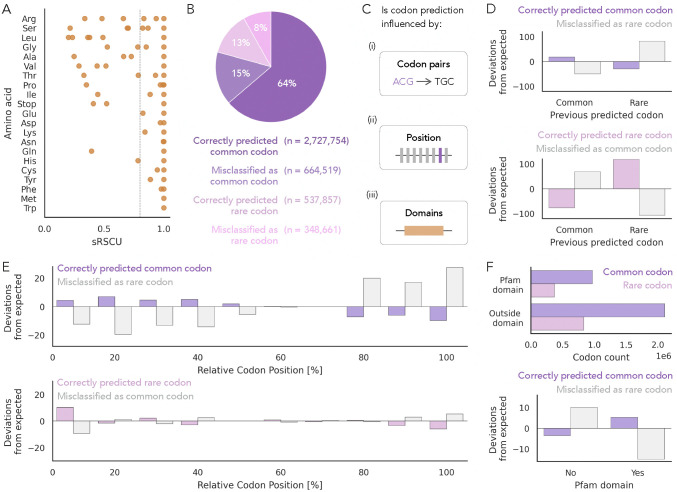
Contextual influences on codon prediction. (A) Rare codons were defined using an sRSCU threshold of <0.8. (B) Predictions for all 4,351,017 codons from ~6,000 human sequences in the test set were categorized into four classes based on whether the predicted codon was common or rare and whether it matched the wild-type codon. (C) To test whether codon predictions are influenced by previous codon identity, sequence position, or domain context, we performed separate chi-squared tests for true common codons (correctly predicted common and misclassified as rare) and true rare codons (correctly predicted rare and misclassified as common). For each analysis, observed prediction frequencies were compared to expected frequencies under the assumption of independence between prediction outcome and the feature. (D–F) Standardized residuals are plotted to show deviations from expected frequencies. (D) Prediction outcomes were significantly associated with the identity of the previous codon (p-value < 1e-5). The strongest deviations were observed for rare codons, which were more likely to follow rare codons than expected. (E) Prediction outcomes varied significantly with codon position (p-value < 1e-5). The largest deviations were observed at the 5′ end (first 40%) and 3′ end (last 30%) for true common codons, and within the first 10% for true rare codons. (F) Over 11,000 Pfam domains were identified using InterProScan across the test set proteins. Codon predictions were significantly affected for true common codons (p-value < 1e-5), but not for true rare codons.

We next identified contexts in which Trias prefers rare codons. Defining rare codons as those with sRSCU < 0.8 (Figure 3A), we classified all 4 million codon predictions from the human test set sequences into four categories based on their wild-type identity and model prediction: (i) correctly predicted common codon, (ii) misclassified as rare codon (common codons incorrectly predicted as rare), (iii) correctly predicted rare codon, and (iv) misclassified as common codon (rare codons incorrectly predicted as common) (Figure 3B). We then tested whether predictions were affected by the preceding codon, codon position, and protein domain localization (Figure 3C).

We found that codon predictions were influenced by the identity of the previously predicted codon (chi-squared test, p-value<1e-5). Rare codons were more likely to follow other rare codons than expected (Figure 3D, Supplementary Figure S8). A similar trend was observed for common codons, where common codon predictions more often followed other common codons, although the effect was less pronounced.

We also found that the codon position, measured across the relative length of each coding sequence, significantly influences the model’s predictions (chi squared test, p < 1e-5; Figure 3E). Common codons misclassified as rare were less frequent near the 5′ end (first 40%) and more frequent toward the 3′ end (last 30%) of sequences. Correctly predicted rare codons were enriched within the first 10% at the 5′ end. This suggests that Trias tends to overestimate rare codon usage toward the end of coding regions, but more accurately predicts rare codons at the beginning.

Finally, we investigated whether codon predictions were affected by location within or outside of protein domains. Around 30% of rare codons and 30% of common codons were located within annotated Pfam domains (Figure 3F). Within domains, common codons were more often predicted as such, and misclassification of common codons as rare was strongly reduced. Outside of domains, common codons were more frequently misclassified as rare. (No significant effect was found for rare codons.) These results suggest that codon usage within domains is more constrained, and Trias more closely aligns with wild-type codon choice for common codons in these regions.

### 2.4 Rare codon context biases generative models toward continued rare codon use

As shown in Figure 2A, Trias-generated sequences generally favor high-frequency codons even for genes whose natural sequence uses rare codons. However, a distinct subset of sequences are generated with rare codons, resulting in codon usage patterns that more closely resemble their wild-type counterparts. This may reflect the tendency of rare codons to cluster in natural sequences (Clarke and Clark, 2024) and is consistent with our earlier observation (Figure 3D) that rare codons are more likely when preceded by other rare codons.

**Figure 4:**
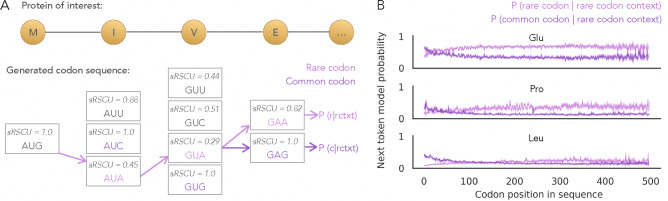
Effect of codon context on next token probabilities in Trias. (A) Next token probability is calculated for selecting the rarest (lowest sRSCU) or most common (highest sRSCU) codon, given that all preceding codons are rare. These are denoted as *P*(*r* | rctxt) and *P*(*c* | rctxt), with rctxt: rare codon context; r: rare codon; c: common codon. (B) Mean probabilities and confidence intervals across ~2,500 human sequences in the test set (sequence length ≤500 codons) are shown for three amino acids: glutamate (Glu), proline (Pro), and leucine (Leu).

To better understand how rare codon context influences predictions, we designed an *in silico* experiment on the human test set sequences (Figure 4A). At each position, we constrained all preceding codons to be rare (lowest sRSCU per amino acid) and evaluated the probability of selecting the next codon as rare versus frequent (highest sRSCU). We hypothesized that Trias would adapt to a history of seeing rare codons by assigning higher probabilities to subsequent rare codons throughout the sequence, even when globally such choices would be suboptimal. Consistent with our hypothesis, the preference for rare codons increased with the length of the preceding rare-codon context (Figure 4B). This trend was observed across all amino acids, with smaller probability differences for amino acids that have more synonymous codons (Supplementary Figure S9). We conclude that the model learned a form of internal consistency: although it is likely to predict preferred codons in most situations, it can detect genes that have stretches of rare codons and continue generating additional rare codons in these sequences.

### 2.5 Trias generates codon sequences that align with experimental data

**Figure 5:**
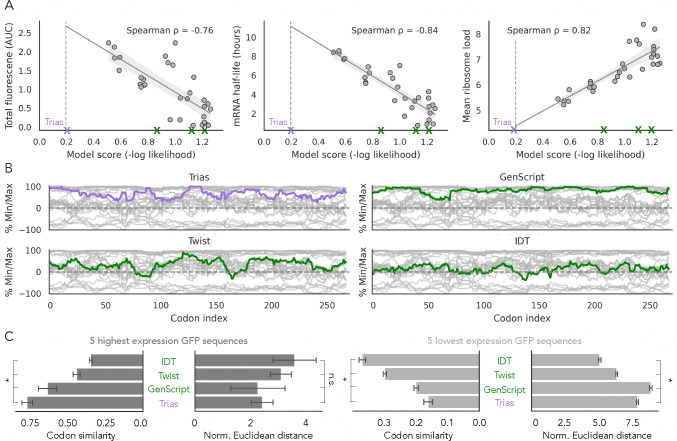
Trias-predicted codon sequences correlate with experimental data and differ from commercial codon optimization tools. (A) Correlation between Trias-predicted sequence likelihood and experimentally measured properties of 30 GFP codon variants expressed in HEK293 cells (data from Bicknell et al. (2024)). The purple cross marks the Trias-generated sequence obtained via greedy decoding (score = 0.19), with the regression line extrapolated up to this point. AUC: area under the curve quantifies total GFP fluorescence over time. (B) %MinMax codon usage profiles of all 30 GFP variants (gray) compared to sequences generated by Trias and three commercial codon optimization tools (GenScript, Twist, IDT). Trias likelihood scores for sequences generated by these tools were 0.86, 1.13, and 1.22 respectively (green markers in A). (C) Codon similarity and normalized Euclidean distances comparing Trias and commercial tool-generated sequences to the five highest- and lowest-expression GFP variants. Asterisks (*) denote statistical significance (p-value<0.05); error bars show standard deviation.

To evaluate the connection between Trias predictions and protein output, we benchmarked its predictions against experimental data. A recent study from Bicknell et al. (2024) measured the output of 30 different encodings of GFP, differing only in their codon composition and spanning a range of sRSCU and MFE values, that were transfected into HEK293 cells as N1-methylpseudouridine-modified GFP mRNAs (Supplementary Figure S10). We scored each sequence with Trias by computing the negative log-likelihood of the model’s probabilities, normalized by sequence length, and tested correlations with experimental measurements (Figure 5A). Trias scores correlated strongly with the measured GFP protein output (*ρ* = −0.76), mRNA half-life (*ρ* = −0.84), and mean ribosome load (*ρ* = 0.82). Despite being trained only on natural sequences without any experimental data, Trias learns codon patterns that align with experimentally measured properties. In contrast, sRSCU alone is insufficient as a predictor (Supplementary Figure S10B), highlighting Trias’ ability to capture additional sequence context beyond codon frequency.

In addition to scoring the experimental GFP variants, we generated a new human GFP codon sequence using a greedy decoding approach. Its likelihood score of 0.20 exceeded the scores of all experimentally tested sequences. Given the strong correlation between model scores and experimental protein expression, we extrapolated the regression line in Figure 5A to estimate a potential expression level of around 2.67 for this Trias-optimized sequence. We also compared this optimal Trias-generated GFP sequence to sequences designed by commercially available codon optimization tools from GenScript^1^, Twist^2^ and IDT^3^. Trias ranked these sequences significantly lower, with likelihood scores of 0.86, 1.13 and 1.22, respectively (Figure 5A). We found that the Trias and GenScript sequences predominantly followed the upper range of the possible %MinMax profiles, indicating a preference for more frequently used codons (Figure 5B). While GenScript consistently chose frequent codons, Trias used a wider range of codon choices, perhaps reflecting more nuanced rules.

We then compared the sequences produced by Trias and commercial codon optimization tools to the highest- and lowest-expressing GFP variants (Figure 5B,C). The Trias-generated sequence showed high codon similarity to the five highest-expressing variants (76%) and low similarly to the lowest-expressing variants (16%). The sequences generated by GenScript, Twist and IDT were significantly less similar to the high expression variants. Similarly, the %MinMax profiles of Trias and GenScript sequences more closely resembled those of the highest-expressing variants, while Twist and IDT matched more closely with mid-sRSCU GFP variants (Supplementary Figure S11). Taken together, these comparisons with empirical results suggest that Trias-generated sequences more effectively capture codon usage patterns linked to high protein expression compared to commercially optimized sequences.

## 3 Discussion

Trias is a species-aware encoder-decoder model trained on 10 million vertebrate coding sequences to reverse translate protein sequences into codon sequences. Trias learns codon usage directly from natural sequences, capturing biologically meaningful patterns beyond simple frequency-based approaches. This data-driven strategy presents a promising alternative for designing codon-optimized mRNA sequences.

Our analysis reveals that Trias favors common codons and generates sequences with high mRNA stability. It also retains rare codons in biologically relevant positions, indicating that it captures functional constraints on codon choice. Gene-specific differences in rare codon usage suggest Trias learns rules beyond general frequency trends.

An implicit assumption in many applications of nucleotide foundation models for sequence design is that higher-likelihood sequences will correspond to higher protein expression. While this may hold in some cases, natural mRNAs are not necessarily optimized for high expression. Despite this conceptual limitation and without explicit training on experimental protein expression data, Trias’ predicted scores for GFP codon variants show strong correlations with protein output, mRNA half-life, and ribosome load. Thus, our results validate the approach of learning from natural optimization in designing synthetic mRNAs for high expression.

The exceptions to this pattern are important to consider. We find that Trias’ autoregressive decoding introduces a form of local internal consistency, where rare codon choices are more likely to be followed by additional rare codons. This behavior can lead to generation of sequences with codon usage that may echo nature but does not correspond to high expression. These findings have broader implications for the use of generative language models in predictive settings. Off-the-shelf models trained on natural sequences may carry biases that affect downstream applications, not only for zero-shot prediction of protein expression, but also for tasks such as variant effect prediction, where sequence likelihoods are often used as proxies for functional impact or pathogenicity. For Trias, fine-tuning on protein expression data could help reduce these biases and further enhance its ability to design codon sequences optimized for high protein expression.

Foundation models of nucleic acid sequence have become promising tools for synthetic mRNA sequence design, with broad applications in recombinant protein production and mRNA-based therapeutics. Unlike commercial codon optimization tools that rely on predefined rules, Trias leverages a data-driven approach that generalizes across species and functional contexts. Our benchmarking demonstrated that Trias-generated sequences more closely resemble high-expression GFP variants than those from commercial tools. In contrast to other deep learning models, Trias focuses on vertebrate sequences to gain specific insight into the rules most likely to apply to human mRNA design; these rules can differ in key ways from prokaryotic translation constraints. Despite training on a smaller, phylogenetically narrower dataset, our model achieves strong performance, supporting the value of models tailored to specific biological scenarios. Trias is distinct in integrating generative sequence design into its architecture, limiting our ability to compare it directly to published codon language models that focus on predictive tasks rather than sequence generation. Nonetheless, our benchmarking against experimental protein expression data demonstrates that Trias effectively captures biologically relevant codon usage patterns that translate to improved protein output.

Ultimately, a complete model for synthetic mRNA design must incorporate regulatory features beyond the codon sequence itself. Untranslated regions (UTRs) play key roles in mRNA stability and translation efficiency (Mayr, 2017; Leppek et al., 2018), and the UTR sequence can affect the impact of codon choice (Lyons et al., 2024). Understanding these interactions could further refine the model’s ability to design synthetic mRNA sequences; recent work has shown that incorporating UTRs can improve predictions of mRNA properties and protein expression (Li et al., 2025). A model of synthetic mRNA must also consider the impact of chemical modifications such as with N1-methylpseudouridine that are necessary to reduce innate immune reactions to therapeutic mRNAs (Metkar et al., 2024). These modifications, not found in natural mRNAs, may affect mRNA structure and translation efficiency. Therefore, fine-tuning Trias on chemically modified sequences will be important to account for these effects and adapt codon choices accordingly. Future work to expand the scope of our model can fully unlock the potential of generative models for mRNA design in synthetic biology and therapeutic applications.

## 4 Methods

### 4.1 Datasets

#### 4.1.1 Training and test dataset

We downloaded all vertebrate sequences from the NCBI RefSeq database^4^ on August 5th, 2024. To ensure data quality, we applied the following filtering criteria: sequences labeled as “pseudo” were excluded, and only records containing the feature tags “CDS” and “translation” were kept. We verified that each sequence was complete, initiated with the start codon “ATG” and terminated with one of the three stop codons (“TAA” “TGA” or “TAG”). Additionally, the translated protein sequences were required to start with methionine “M” and end with a stop symbol “*”.

After processing the downloaded records, we filter the mRNA sequences according to the length of the coding region. This step resulted in approximately 26 million sequences. To reduce redundancy, we clustered sequences with 90% or greater nucleotide similarity using MMSeqs2 and kept only one representative sequence per cluster. This step reduced the data set to around 10.2 million sequences.

To evaluate model performance, we created a train-test split from our curated dataset of 10.2 million coding sequences. For the test set, we randomly selected 300 human genes from each expression category (high, medium, and low) based on GTEx expression levels, ensuring a diverse range of expression profiles. These 900 genes were translated into protein sequences and used to query the rest of the dataset using BLAST. Any sequences in the remaining dataset with significant protein similarity to these test genes were removed and added to the test set to avoid overlap. This resulted in a test set of approximately 200,000 sequences, including the original 900 human genes. In total, the test set contains approximately 6,000 human coding sequences, including multiple splice isoforms of some genes. The final training set consisted of all 10 million remaining sequences from the curated dataset after removing those with significant protein similarity to the test genes. This approach ensured that the test set, used for performance evaluation, did not include sequences with close homologues in the training set. To monitor training progress, we randomly sampled 1% of the training data as a validation set, with sampling balanced across species.

#### 4.1.2 Gene expression data from GTEx

The GTEx dataset provides gene expression data across 54 human tissues from approximately 1,000 individuals, measured via bulk RNA sequencing (RNA-seq). For this study, gene expression data were obtained from the GTEx Portal^5^ on December 11th 2024, from the GTEx_Analysis_2017-06-05_v8_RNASeQCv1.1.9_gene_median_tpm.gct.gz file, which contains median gene-level TPM values per tissue. Genes with no expression (TPM = 0) in any tissue were excluded. We then computed the median TPM across all tissues and retained only genes that matched those in our human test set.

### 4.2 Model architecture and training

Trias is based on the HuggingFace implementation of the BART encoder-decoder architecture (Lewis et al., 2019). The input sequence length is set to 2,048 tokens, with the first token reserved for a species tag to ensure species-specific optimization. The vocabulary consists of 64 codon tokens, 20 amino acid tokens, 640 species-specific tokens, and 4 special tokens (</s>, <unk>, <pad>, <mask>).

The model uses the BartForConditionalGeneration class, with 15% of amino acid tokens in the protein sequence masked during training. Flash attention is applied to improve memory efficiency and computational speed. Optimization is performed using the Adam optimizer with a cosine learning rate schedule and warm-up steps. The learning rate starts at 1e-4 and a weight decay of 0.01 is applied. Dropout is set to 0.1. The model is trained for 650,000 steps, corresponding to approximately two epochs.

Training is carried out on an NVIDIA A100 GPU with 80 GB of memory. The batch size is set to 8, and gradient accumulation is used with an accumulation factor of 4, resulting in an effective batch size of 32. The architecture consists of 6 encoder layers and 6 decoder layers, each with 8 attention heads. The hidden size of the feedforward networks is 2,048 for both the encoder and decoder. This configuration results in a model with approximately 47M trainable parameters.

### 4.3 Data analysis and performance metrics

#### 4.3.1 Gene ontology enrichment analysis

To investigate functional constraints on codon usage, we performed a gene ontology (GO) enrichment analysis on genes with predicted low and high sRSCU values. Prior to analysis, we removed isoforms from the human test set, resulting in a non-redundant set of 879 genes (down from 6,165 sequences, including isoforms). For each gene, we aggregated predicted and natural sRSCU values by taking the mean across isoforms. We classified genes into two groups based on their predicted sRSCU: genes with low sRSCU (≤0.86) and high sRSCU (>0.86). We then performed GO analysis to identify functional terms overrepresented in these subsets compared to the full set of 879 human test genes. GO term enrichment was assessed using the gprofiler Python package, with the background set defined as all 879 genes in the test set. We applied Bonferroni correction for multiple hypothesis testing and set a significance threshold of p-value < 0.05. GO terms related to molecular function, biological process, and cellular component categories were considered in the analysis.

#### 4.3.2 Codon usage calculation

To quantify codon usage bias, we computed the scaled relative synonymous codon usage (sRSCU) for every codon in each species based on species-specific codon frequency in the training set. sRSCU normalizes codon frequency within synonymous codon categories, allowing direct comparisons across different amino acids. It measures how frequently a given codon is used relative to the expected uniform distribution of synonymous codons for an amino acid. The relative synonymous codon usage (RSCU) for a given codon c is calculated following Sharp et al. (1986):

(1) $${RSCU}_{i,j}=\frac{c_{i,j}}{\frac{1}{n_{i}}\sum_{j=1}^{n_{i}}\;c_{i,j}},$$

where $c_{i,j}$ is the observed frequency of the jth codon for the ith amino acid in the training set for a specific species, and $n_{i}$ is the number of synonymous codons for the ith amino acid. To derive sRSCU, we scale RSCU values by dividing by the highest RSCU value for each amino acid within a species:

(2) $${sRSCU}_{i,j}=\frac{{RSCU}_{i,j}}{max\left({RSCU}_{i,1},{RSCU}_{i,2},\ldots ,{RSCU}_{i,n_{i}}\right)}$$

This ensures that the most frequently used synonymous codon receives a score of 1, while other synonymous codons are scaled accordingly. To calculate the sRSCU for a gene, we computed the average sRSCU value of all codons within that gene.

#### 4.3.3 Expected value calculation for chi-squared tests

We used chi-squared tests to assess whether prediction outcomes were associated with specific sequence features (previous codon, codon position, and domain localization). Codon predictions from the test set were categorized into four classes (correctly predicted common, misclassified as rare, correctly predicted rare, or misclassified as common). Observed frequencies for all four prediction categories across each sequence feature are reported in Supplementary Tables S1–S4. For the chi-squared tests, we constructed separate contingency tables for true common codons (correctly predicted common and misclassified as rare) and true rare codons (correctly predicted rare and misclassified as common). Expected counts were calculated under the null hypothesis that codon prediction categories are independent of the tested feature. Expected values $E_{ij}$ were computed as follows:

(3) $$E_{ij}=\frac{R_{i}\;\cdot \;C_{j}}{N},$$

where $R_{i}$ is the total count for feature level $i,\;C_{j}$ is the total count for prediction category j, and N is the total number of codon predictions. To identify which feature levels contributed most to significant associations, we also computed standardized residuals.

#### 4.3.4 Pfam domain annotation

We used InterProScan to identify protein domains in our test set. It scans protein sequences against a comprehensive collection of databases to annotate known domains, families, and functional sites. InterProScan was run locally on all ~6,000 human protein sequences in the test set. From the resulting annotations, we extracted Pfam domain matches and kept only those with e-values <1e-5 to ensure high-confidence predictions. The e-value indicates the number of matches expected by chance, with lower values reflecting higher significance. Codons were labeled as within a domain if they overlapped a detected Pfam domain, and as outside a domain otherwise.

#### 4.3.5 Codon usage profiles

To analyze codon usage variability along sequences, we computed %MinMax profiles, which quantify deviations in codon usage relative to expected frequency distributions. This approach is adapted from Rodriguez et al. (2018) and provides a measure of codon bias by comparing observed codon frequencies within a sliding window (size = 18) to the expected frequency range. Positive %MinMax values indicate a preference for frequently used codons, while negative values reflect increased usage of rare codons.

To compare codon usage patterns between sequences, we used normalized Euclidean distance between their %MinMax profiles. The Euclidean distance was computed across the profile and normalized by sequence length. This approach provides a quantitative measure of similarity in codon usage fluctuations along the sequence.

#### 4.3.6 Sequence likelihood scoring

To assess the likelihood of a codon sequence S, we define a scoring function L(S) based on the negative log-likelihood assigned by the model. Trias decodes codon sequences autoregressively, generating codons one at a time, where each codon is predicted based on the previously generated codons and the input protein sequence. The likelihood of a sequence $S=\left(c_{1},c_{2},\ldots ,c_{N}\right)$ is given by:

(4) $$L\left(S\right)=-\frac{1}{N}\sum_{i=1}^{N}\log P\left(c_{i}|c_{1},c_{2},\ldots ,c_{i-1}\right),$$

where $P\left(c_{i}|c_{1},c_{2},\ldots ,c_{i-1}\right)$ is the model’s predicted probability for codon $c_{i}$, given the preceding sequence ($c_{1},c_{2},\ldots ,c_{i-1}$). The log-likelihood is normalized by sequence length N. A lower L(S) value indicates a sequence that is more likely under the model, meaning the model assigns high probabilities to codon choices at each position. A score of 0 corresponds to a sequence where the model is fully confident in every prediction $\left(P\left(c_{i}\right)=1\right)$, while higher scores reflect more uncertain predictions. Trias uses greedy decoding to generate codon sequences, a decoding strategy in which at each step the model selects the most probable codon. Greedy decoding ensures high-likelihood outputs.

## Supplementary Material

Supplement 1

## Footnotes

1
https://www.genscript.com/gensmart-free-gene-codon-optimization.html

2
https://www.twistbioscience.com/resources/digital-tools/codon-optimization-tool

3
https://www.idtdna.com/pages/tools/codon-optimization-tool

4
https://ftp.ncbi.nlm.nih.gov/refseq/release/

5
https://www.gtexportal.org/home/
