# Supplementary material for: A generative language model decodes contextual constraints on codon choice for mRNA design
Source: bioRxiv. 2025 May 13:2025.05.13.653614. Preprint. [Version 1] doi: 10.1101/2025.05.13.653614 (PMC12132368; doi:10.1101/2025.05.13.653614)
Supplement: Supplementary file 1 [file NIHPP2025.05.13.653614v1-supplement-1.pdf]

bioRxiv preprint doi: https://doi.org/10.1101/2025.05.13.653614; this version posted May 13, 2025. The copyright holder for this preprint (which was not certified by peer review) is the author/funder, who has granted bioRxiv a license to display the preprint in perpetuity. It is made available under aCC-BY 4.0 International license.

SUPPLEMENT

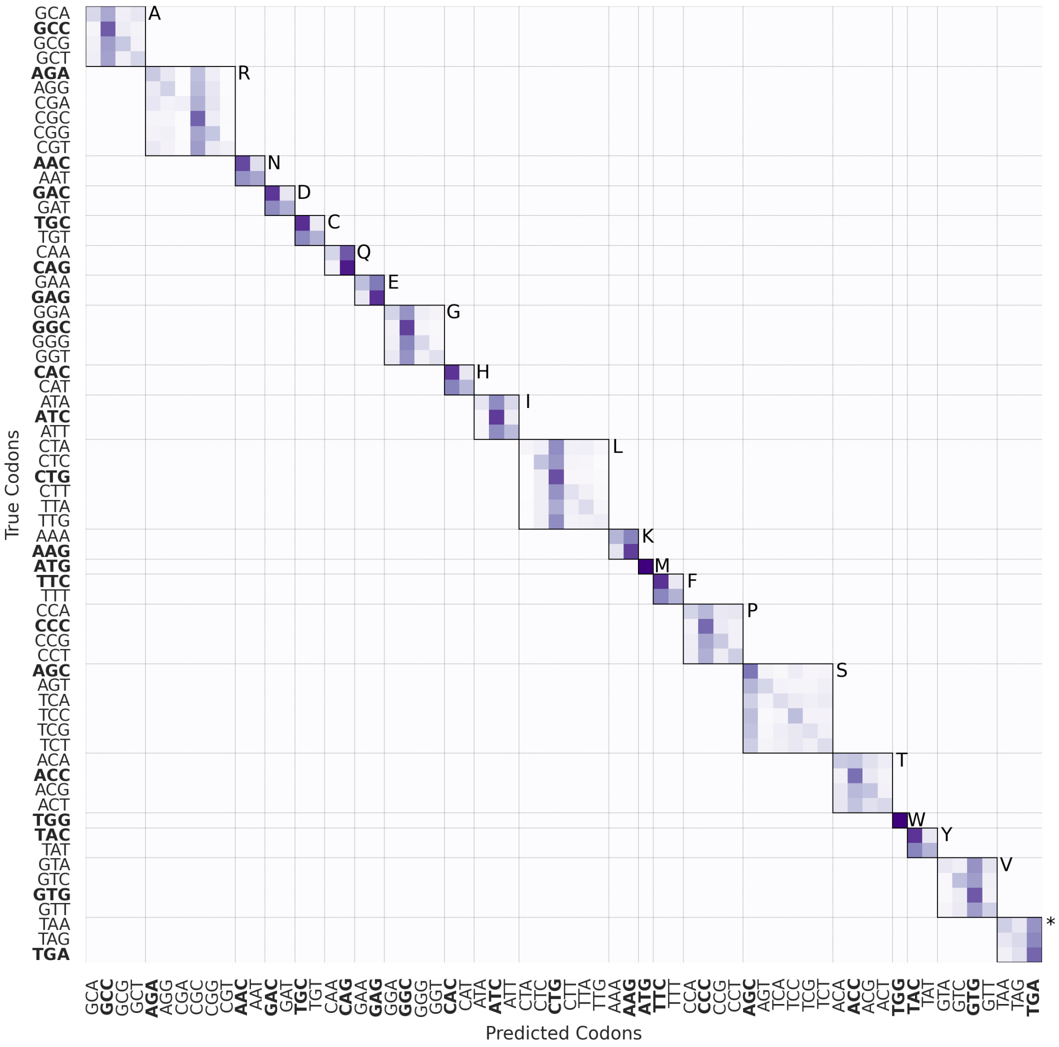

Figure S1: Percentage of Trias-predicted codons matching wild-type. Results are based on 4,351,017 codons from ∼6,000 human test set sequences. Bold codons indicate the most frequent codon per amino acid, with synonymous codons grouped in boxes. The one-letter amino acid code is displayed.

bioRxiv preprint doi: https://doi.org/10.1101/2025.05.13.653614; this version posted May 13, 2025. The copyright holder for this preprint (which was not certified by peer review) is the author/funder, who has granted bioRxiv a license to display the preprint in perpetuity. It is made available under aCC-BY 4.0 International license.

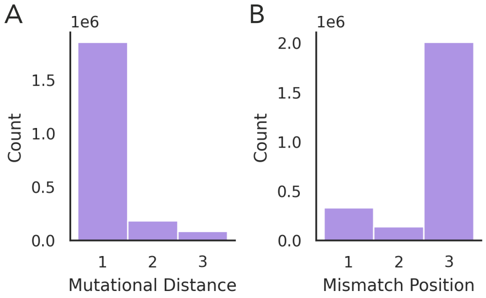

Figure S2: Codon mismatches and their positions. Among 4,351,017 codons in the human test set, approximately half differ from the wild-type codon, while only 0.002% result in an incorrect amino acid. (A) The majority of mismatches involve a single nucleotide substitution. (B) These substitutions predominantly occur at the third codon position.

bioRxiv preprint doi: https://doi.org/10.1101/2025.05.13.653614; this version posted May 13, 2025. The copyright holder for this preprint (which was not certified by peer review) is the author/funder, who has granted bioRxiv a license to display the preprint in perpetuity. It is made available under aCC-BY 4.0 International license.

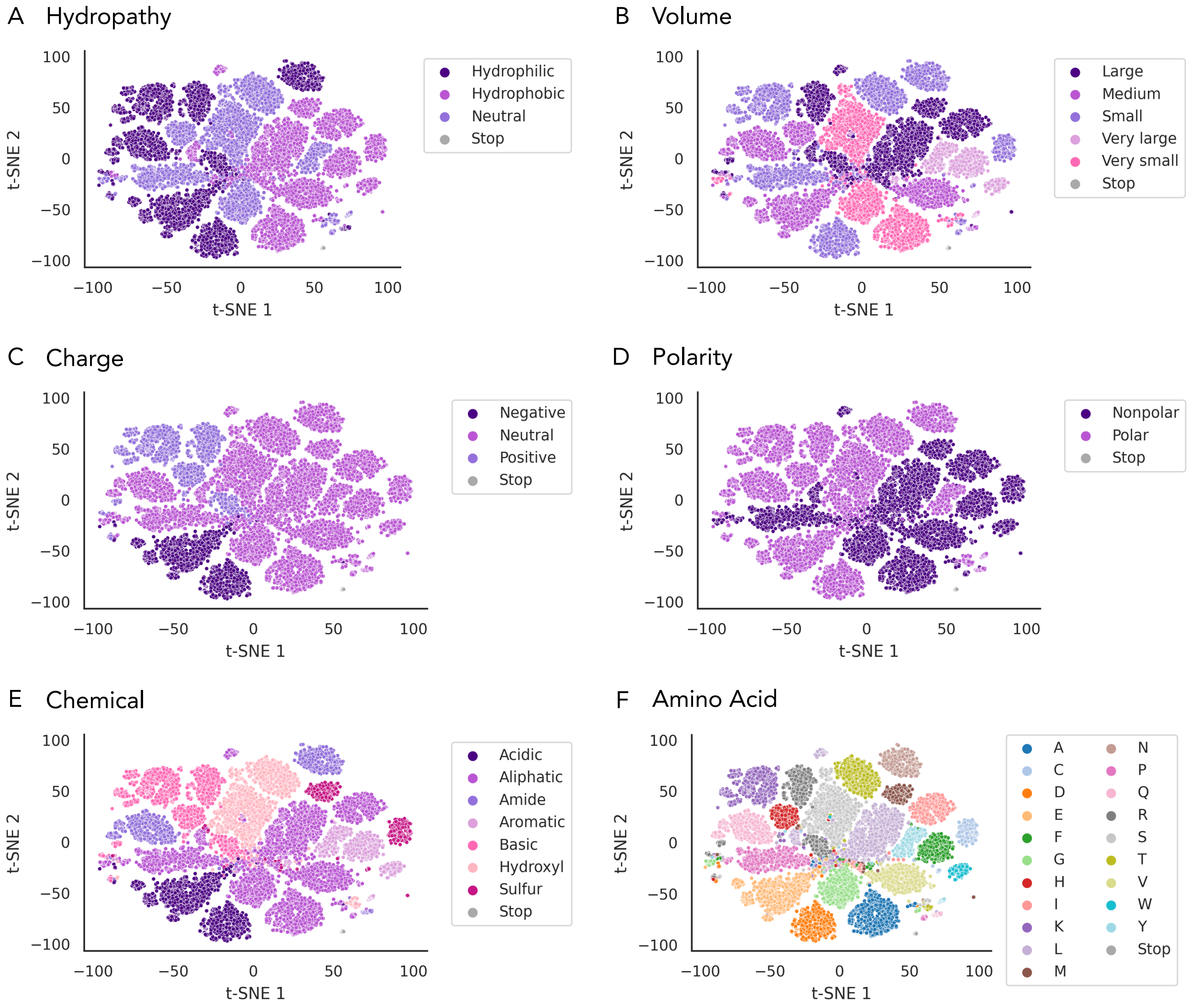

Figure S3: Projection of final encoder embeddings with different labels. Each dot represents an amino acid in 20 randomly selected protein sequences from the human test set. The same projection is shown with different labels based on classifications from this reference table[6].

[6] https://www.imgt.org/IMGTeducation/Aide-memoire/_UK/aminoacids/IMGTclasses.html

bioRxiv preprint doi: https://doi.org/10.1101/2025.05.13.653614; this version posted May 13, 2025. The copyright holder for this preprint (which was not certified by peer review) is the author/funder, who has granted bioRxiv a license to display the preprint in perpetuity. It is made available under aCC-BY 4.0 International license.

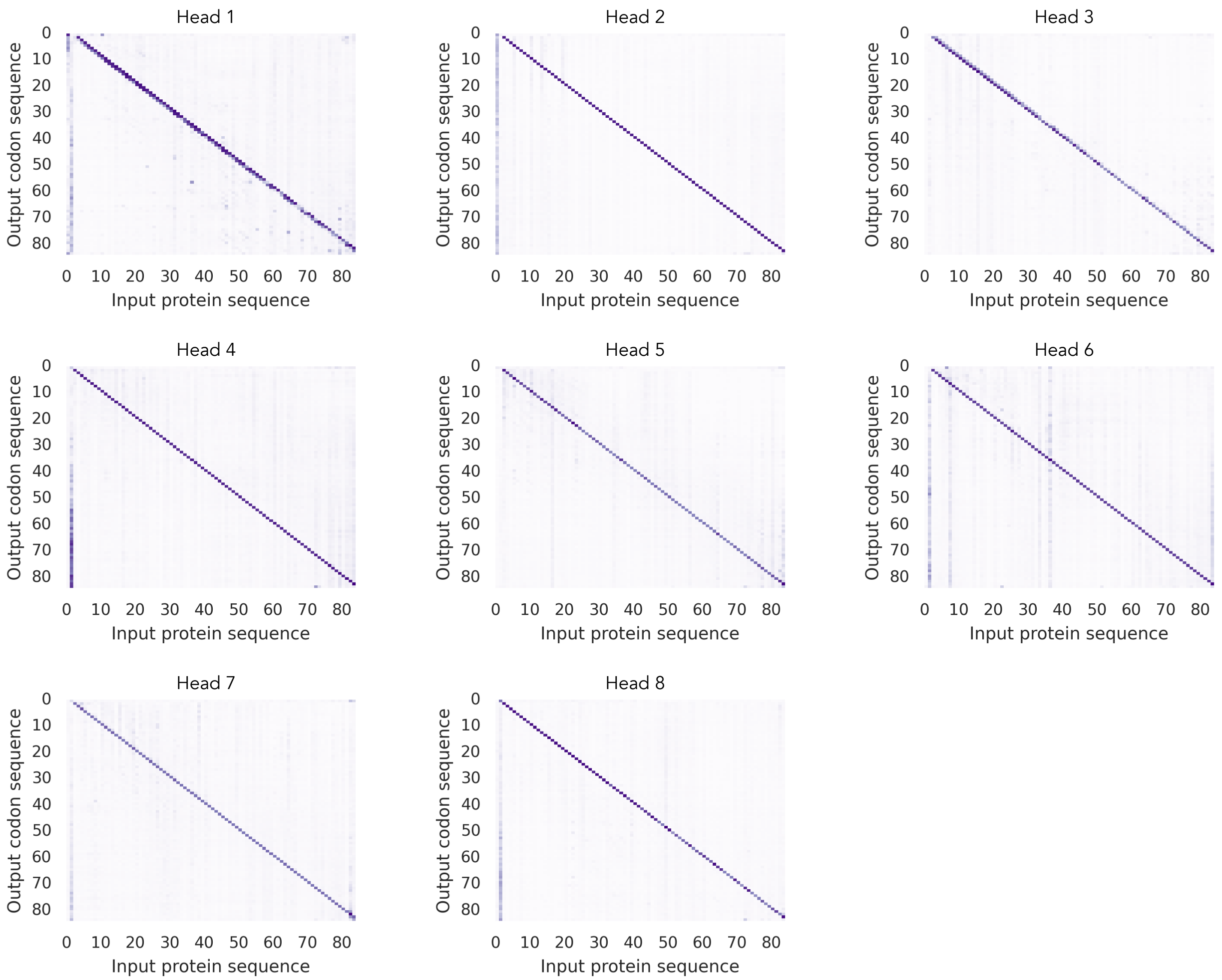

Figure S4: Cross-attention maps for all decoder heads averaged for COX7B2. Each panel shows attention from output codons (y-axis) to input amino acids (x-axis) for one decoder head. Attention weights are averaged across all decoder layers.

bioRxiv preprint doi: https://doi.org/10.1101/2025.05.13.653614; this version posted May 13, 2025. The copyright holder for this preprint (which was not certified by peer review) is the author/funder, who has granted bioRxiv a license to display the preprint in perpetuity. It is made available under aCC-BY 4.0 International license.

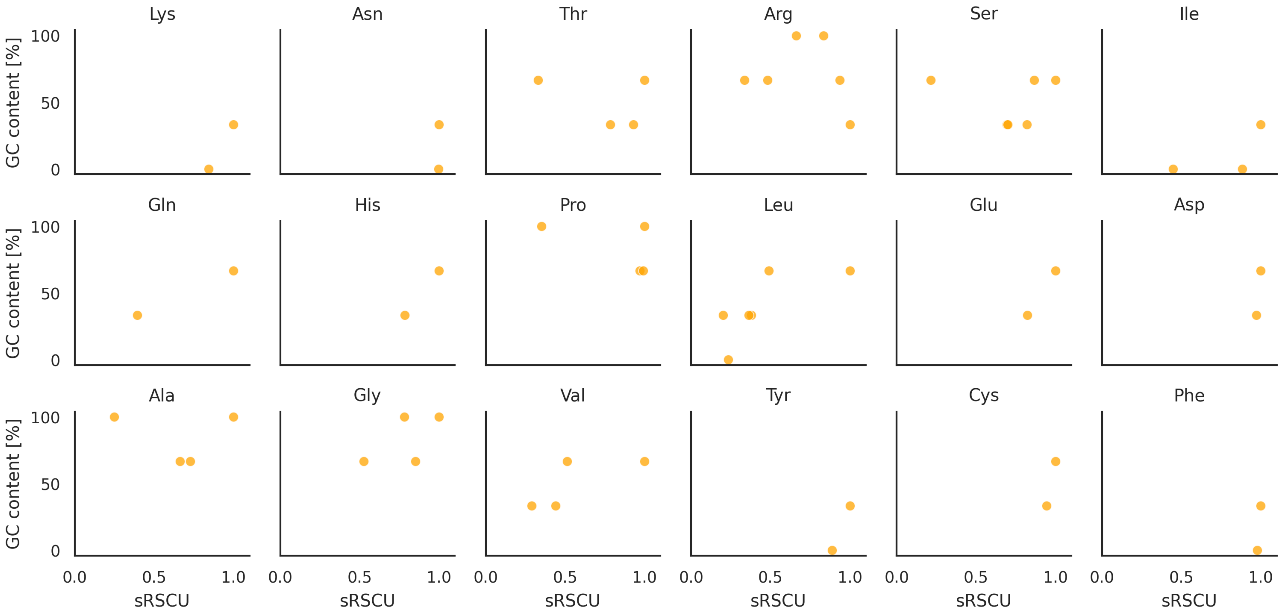

Figure S5: Relationship between codon GC content and sRSCU. For each amino acid, codons were plotted according to their GC content and sRSCU value in humans.

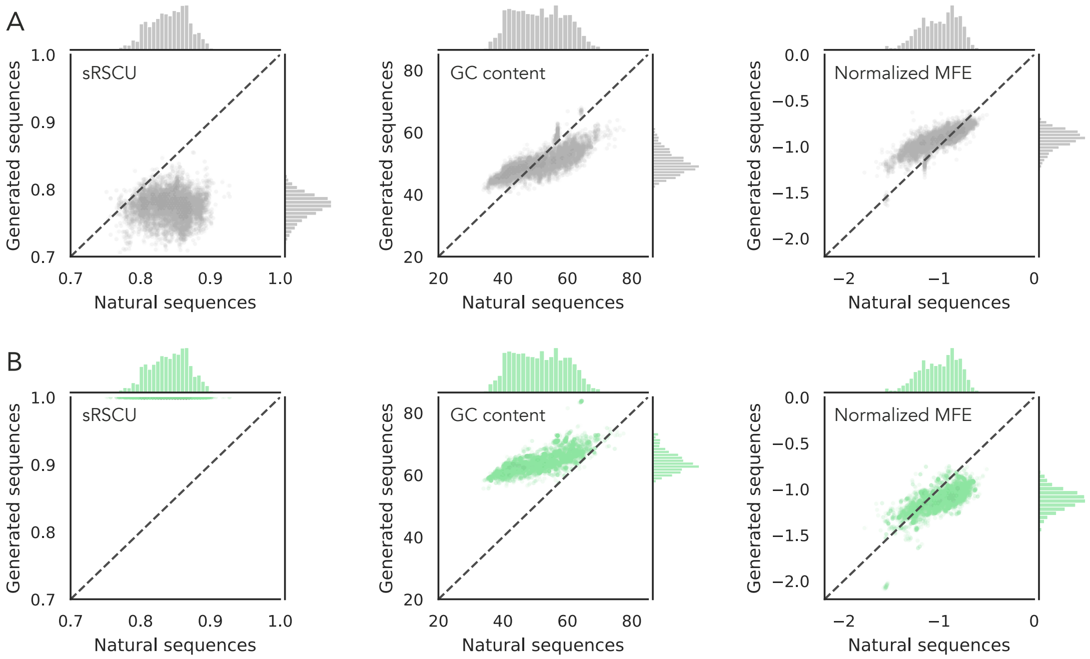

Figure S6: Comparison of generated sequences from a random model (A) and a frequency-based model (B) to their wild-type counterparts across scaled relative synonymous codon usage (sRSCU), GC content, and minimum free energy (MFE, normalized by sequence length) in the human test set (∼6,000 sequences, including isoforms). The random model selects a random synonymous codon for every amino acid site and the frequency-based model selects for every site the most frequent codon.

bioRxiv preprint doi: https://doi.org/10.1101/2025.05.13.653614; this version posted May 13, 2025. The copyright holder for this preprint (which was not certified by peer review) is the author/funder, who has granted bioRxiv a license to display the preprint in perpetuity. It is made available under aCC-BY 4.0 International license.

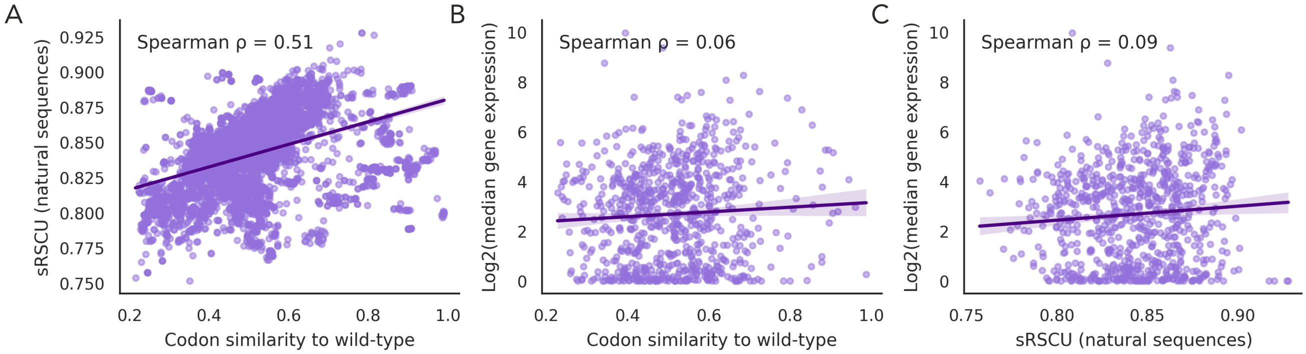

Figure S7: Codon similarity between Trias-generated and wild-type sequences plotted against wild-type sRSCU values for all ∼6,000 human test set sequences (A). Log2-transformed median gene expression across all tissues in the GTEx dataset plotted against codon similarity between Trias-generated sequences and wild-type sequences (B) and against wild-type sRSCU values (C) for 810 human genes in our test set that matched GTEx entries.

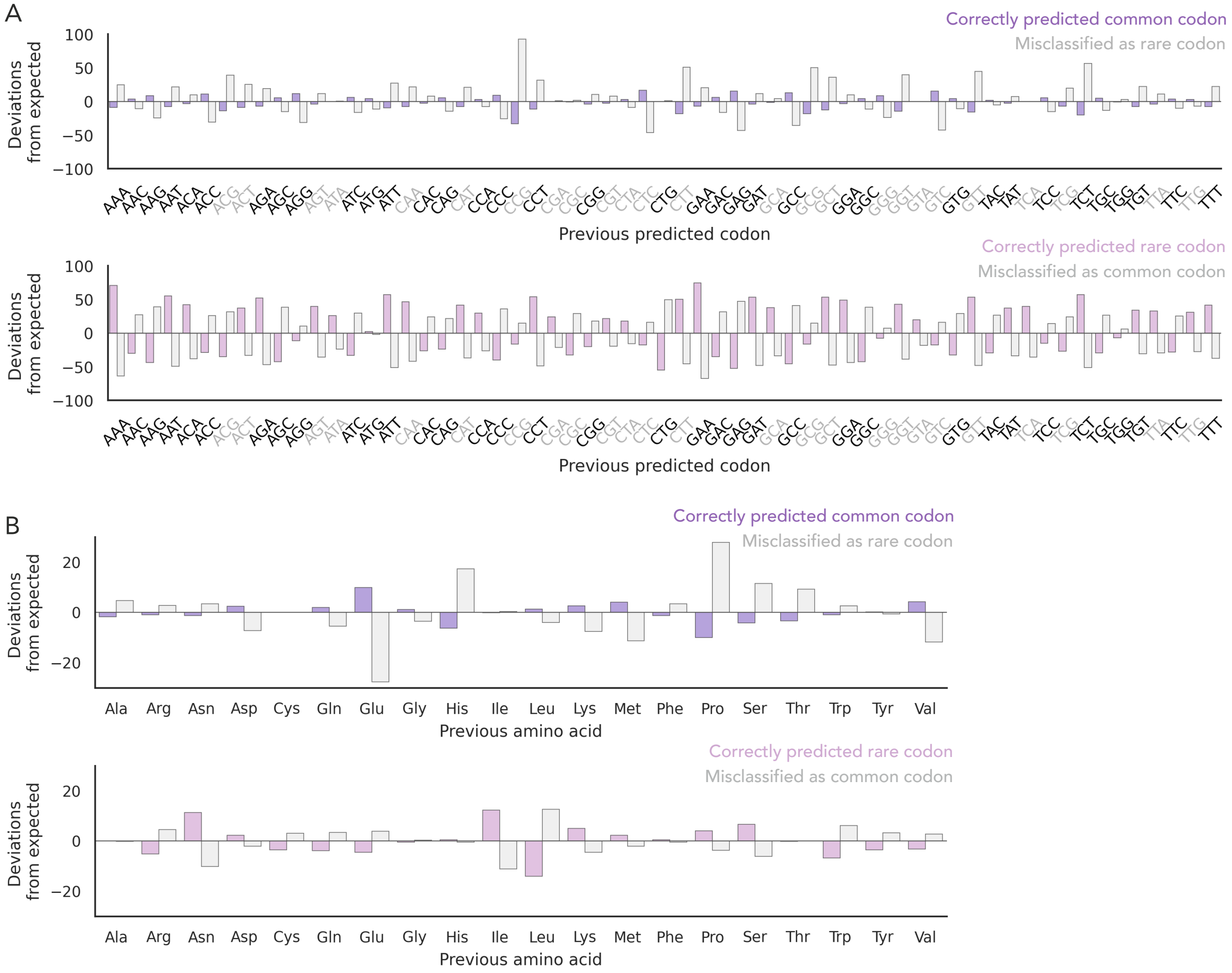

Figure S8: Influence of previous codon or amino acid on codon prediction. (A) Standardized residuals are plotted to show deviations from expected frequencies of next codon prediction classes based on the identity of the previously predicted codon. Rare codons are shown in gray, and common codons in black. (B) Same analysis as in (A), but grouped by the identity of the preceding amino acid.

bioRxiv preprint doi: https://doi.org/10.1101/2025.05.13.653614; this version posted May 13, 2025. The copyright holder for this preprint (which was not certified by peer review) is the author/funder, who has granted bioRxiv a license to display the preprint in perpetuity. It is made available under aCC-BY 4.0 International license.

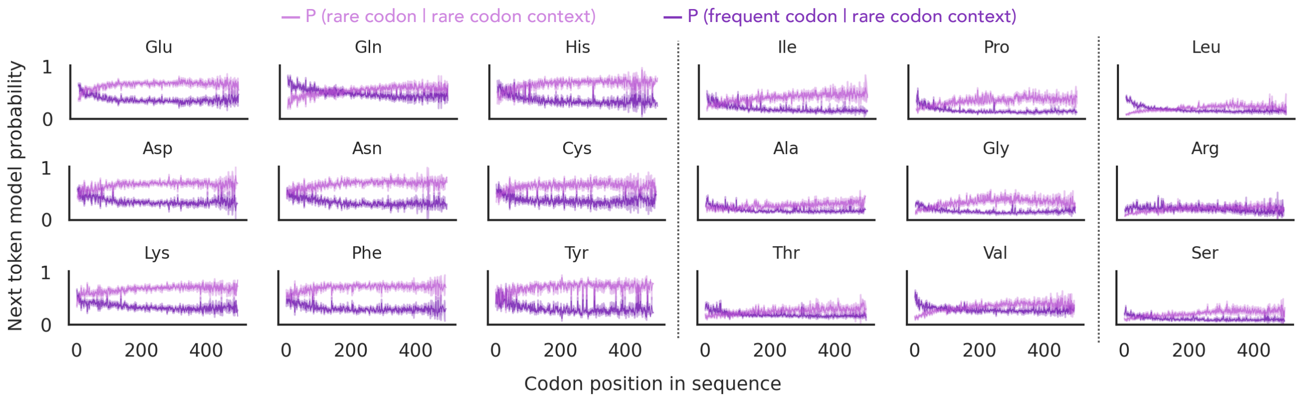

Figure S9: Rare codon context biases next token probabilities across amino acids. Model probability of selecting the next codon as frequent (highest sRSCU) or rare (lowest sRSCU), given that all preceding codons are rare. Amino acids are grouped by the number of synonymous codons (2, 3–4, and 6), with vertical dashed lines marking group boundaries. Mean and confidence interval are shown for ∼2,500 human test set sequences (sequence length ≤500 codons).

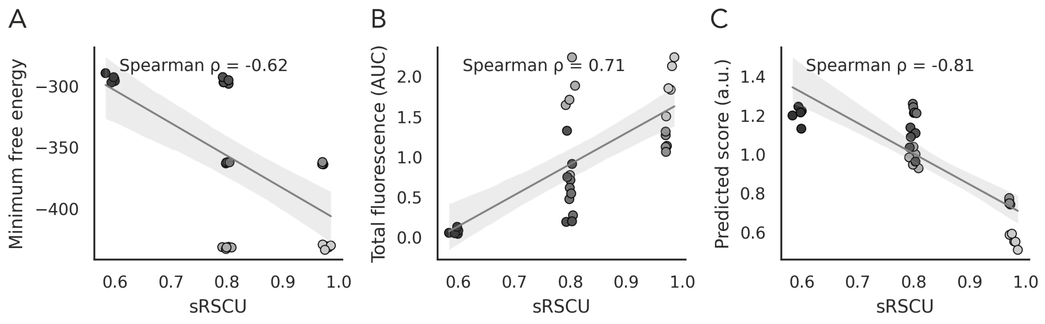

Figure S10: GFP codon variants from Bicknell *et al.* (2024). (A) Minimum free energy (MFE) and codon optimality (sRSCU) values for 30 GFP variants. Variants were selected based on a grid of three distinct sRSCU and three different MFE values, with five variants chosen per region. Different shades of gray indicate the selection regions based on MFE and sRSCU. (B) The same 30 variants, with sRSCU plotted against total GFP fluorescence, quantified as the area under the curve (AUC) from fluorescence measurements in HEK293 cells. (C) sRSCU values plotted against scores predicted by Trias for each of the 30 GFP variants, based on the negative sequence likelihood.

bioRxiv preprint doi: https://doi.org/10.1101/2025.05.13.653614; this version posted May 13, 2025. The copyright holder for this preprint (which was not certified by peer review) is the author/funder, who has granted bioRxiv a license to display the preprint in perpetuity. It is made available under aCC-BY 4.0 International license.

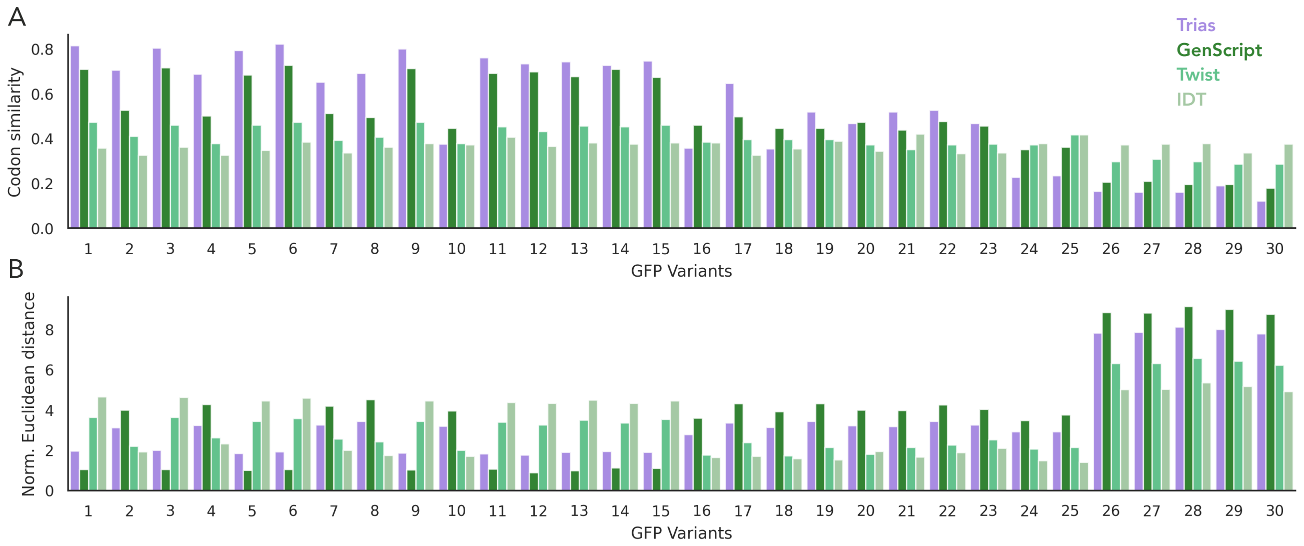

Figure S11: Comparison of Trias and commercial tool sequences to experimentally validated GFP Variants. Codon similarity and normalized Euclidean distances between sequences generated by Trias or commercial tools (GenScript, Twist, IDT) and all 30 GFP variants, sorted by experimentally measured protein expression (variant 1 = highest expression, variant 30 = lowest).

bioRxiv preprint doi: https://doi.org/10.1101/2025.05.13.653614; this version posted May 13, 2025. The copyright holder for this preprint (which was not certified by peer review) is the author/funder, who has granted bioRxiv a license to display the preprint in perpetuity. It is made available under aCC-BY 4.0 International license.

Prev. codon category	Correctly predicted common codon	Misclassified as rare codon	Correctly predicted rare codon	Misclassified as common codon
Common	1,061,767	260,437	393,287	541,552
Rare	260,443	87,260	143,166	121,303

Table S1: Observed prediction counts by codon pair context. Counts of prediction categories for the second codon in each codon pair, grouped by whether the preceding predicted codon was rare or common.

Amino acid	Correctly predicted common codon	Misclassified as rare codon	Correctly predicted rare codon	Misclassified as common codon
Gln	145,099	13,760	19,167	50,557
His	48,976	9,996	19,905	29,988
Ile	131,475	7,205	7,136	32,271
Ala	74,091	29,103	97,426	85,737
Gly	135,556	24,055	35,274	78,994
Pro	219,287	38,118	8,734	18,139
Val	76,757	28,831	69,577	69,098
Thr	102,188	42,316	40,215	46,668
Arg	96,149	69,136	50,250	27,534
Ser	184,841	50,159	62,205	86,616
Leu	107,792	35,009	126,564	137,253

Table S2: Observed prediction counts by amino acid. Counts of prediction categories grouped by the identity of the encoded amino acid. Only amino acids with both rare and common codons are included.

Bin	Correctly predicted common codon	Misclassified as rare codon	Correctly predicted rare codon	Misclassified as common codon
1	269,652	31,902	57,671	65,883
2	273,384	30,882	55,089	68,889
3	273,852	32,251	54,818	66,539
4	276,504	32,329	52,767	66,672
5	274,536	33,939	53,790	66,453
6	274,820	35,057	52,564	64,523
7	273,940	34,997	52,998	65,705
8	270,057	38,732	53,507	65,796
9	270,812	38,227	52,281	66,292
10	270,197	40,345	52,372	67,767

Table S3: Observed prediction counts by relative sequence position. Counts of prediction categories across ten bins representing relative coding sequence positions (each bin corresponds to 10% of the total sequence length).

bioRxiv preprint doi: https://doi.org/10.1101/2025.05.13.653614; this version posted May 13, 2025. The copyright holder for this preprint (which was not certified by peer review) is the author/funder, who has granted bioRxiv a license to display the preprint in perpetuity. It is made available under aCC-BY 4.0 International license.

	Correctly predicted common codon	Misclassified as rare codon	Correctly predicted rare codon	Misclassified as common codon
Pfam domain	1,863,628	243,789	371,947	459,143
Other	864,126	104,872	165,910	205,376

Table S4: Observed prediction counts by domain localization. Counts of prediction categories grouped by whether the codon is located within or outside an annotated Pfam domain.
